# Mapping global epidemiology of thyroid nodules among general population: A systematic review and meta-analysis

**DOI:** 10.3389/fonc.2022.1029926

**Published:** 2022-11-10

**Authors:** Chunyang Mu, Xin Ming, Ye Tian, Yang Liu, Menglin Yao, Yinyun Ni, Yong Liu, Zhihui Li

**Affiliations:** ^1^ Department of Liver Surgery and Liver Transplantation Center, West China Hospital, Sichuan University, Chengdu, China; ^2^ Department of Ultrasound, West China Hospital, Sichuan University, Chengdu, Sichuan, China; ^3^ Department of Obstetrics and Gynecology, Second Affiliated Hospital, Chongqing Medical University, Chongqing, China; ^4^ Department of Respiratory and Critical Care Medicine, National Clinical Research Center for Geriatrics, Center of Precision Medicine, Precision Medicine Key Laboratory of Sichuan Province, Frontiers Science Center for Disease-related Molecular Network, West China Hospital, West China School of Medicine, Sichuan University, Chengdu, Sichuan, China; ^5^ Department of Gastroenterological Surgery, West China Hospital, Sichuan University, Chengdu, China; ^6^ Department of Thyroid and Parathyroid Surgery, West China Hospital, Sichuan University, Chengdu, Sichuan, China; ^7^ Laboratory of Thyroid and Parathyroid Diseases, Frontiers Science Center for Disease-Related Molecular Network, West China Hospital, Sichuan University, Chengdu, Sichuan, China

**Keywords:** epidemiology, thyroid nodule, prevalence, Ultrasound, Autopsy

## Abstract

**Introduction:**

An emerging public health issue is brought on by the worldwide increase of thyroid nodules (TNs). The goal of the current study is to determine the global prevalence of TNs among the general population.

**Methods:**

We screened articles published from January 2000 to May 2022. TN prevalence was calculated with the DerSimonian–Laird random effects model with arcsine transformation.

**Results:**

A total of 20,358 entries were found in our research, and 102 of them met our inclusion criteria. A total of 9,276,178 individuals have been diagnosed as TNs; the overall prevalence was 24.83% (95% CI 21.44–28.55), regardless of the diagnostic techniques. TNs have become more prevalent during 2012–2022 (29.29%) compared with 2000–2011 (21.53%, *p* = 0.02). In addition, we discovered that women (36.51%) were more likely to have TNs than men (23.47%, *p* < 0.01). Interestingly, we found that obesity was correlated with the prevalence of TNs. Additionally, age-specific-stratified TN prevalence was found in our results.

**Discussion:**

This meta-analysis shows that, regardless of country development and economic status, TNs are spreading more widely over the world. Our findings showed a strong correlation between rising TN prevalence and older age, female sex, and elevated weight. To stop the TN epidemic from spreading over the world, increased awareness, the understanding of the disease, and quick action are required.

## Introduction

Thyroid nodules (TNs) are lesions inside the thyroid gland that are radiologically different from the surrounding thyroid parenchyma (1). TNs are common and becoming more prevalent in clinical practice. Their prevalence in the general population varies from 2 to 65% depending on diagnostic techniques (2). They are discovered either clinically on self-palpation by a patient, during a health checkup by a clinician, or incidentally during a radiologic procedure such as ultrasonography (US) imaging, computed tomography (CT), or magnetic resonance imaging (MRI) of the neck, or fluorodeoxyglucose (FDG) positron emission tomography for other indications. With the increased use of sensitive imaging techniques, TNs are being diagnosed incidentally with increasing frequency in the recent years (3). To be mentioned, the diagnosis of TNs includes a fraction that harbored considerable malignancy (approximately 10%) and those that cause (or are at danger of causing) compressive symptoms (5%) or those who have thyroid dysfunction (5%), and these findings are clinically meaningful (4). Although there is little evidence available about how iodine influences the natural history of TNs, the relationship between iodine intake and the occurrence of TNs has been the subject of numerous prior studies (5). There has been a lot of focus on TN risk factors. Advanced age, female gender, and obesity have been reported to increase the susceptibility for TN (6, 7). In addition, it has also been asserted that the metabolic syndrome and its components are associated with TN prevalence (8, 9). Moreover, estrogen dominance can cause TNs to develop or can be a contributing factor (10, 11). To be noted, TNs are more common in countries with iodine-deficient populations (12). Although the introduction of iodized salt virtually eliminated iodine deficiency disorders across the globe, the real prevalence of TNs is largely unknown. Therefore, we perform current study in order to map the global epidemiology of TNs and to investigate whether the TNs prevalence changed by age, gender, country development, and economic status.

## Methods

### Study selection

A systematic review and meta-analysis was carried out in accordance with the Meta-Analysis of Observational Studies in Epidemiology guidelines. From the beginning to May 2022, a thorough search was conducted in PubMed, Embase, and Web of Science for studies that reported the prevalence of TNs in the general population, defined as individuals without a known history of thyroid disease or thyroid cancer treatment, as well as studies of populations that had not been exposed to radiation (i.e., Hiroshima and Nagasaki atomic bombings and Chernobyl nuclear power plant accident). Our inclusion criteria were as follows (1): TNs detected by imaging (ultrasound, CT, or MRI/spectroscopy), biopsy, or autopsy and (2) the study provided information on disease prevalence. Studies were excluded as follows (1): the study was a review article, abstract, case report, correspondence, conference paper, or meta-analysis (2); did not identify individuals with TNs (3); individuals < 18 years (4); studies that reported atomic bomb or Chernobyl nuclear disaster survivors (5); study period was not during January 2000 to May 2022 (6); TNs diagnosed by palpation; and (7) no sufficient information for data extraction.

Two authors (Y. Tian and X. Ming) independently determined whether studies were suitable, evaluated the methodological soundness of the candidate studies, and gathered data using forms created specifically for this study. The authors settled discrepancies by jointly inspecting the study being referred to. On the off chance that no accord was reached, a third author (Z. Li), who was not informed of the previous rulings, served as an arbitrator.

### Data extraction

Two authors (Y. Tian and X. Ming) independently reviewed and extracted data from the included studies by utilizing the data extract form. The duplicate with the smallest sample size or shortest follow-up period was removed when duplicate data were found. The prevalence of TNs in the general population was the most crucial factor for this meta-analysis. In addition, we pooled estimate of prevalence in subgroups, including different countries, country development, and country income assessed by World Bank, study time, study size, study quality score, body mass index, and diagnostic techniques. In addition, we also collected the data about the characteristics of the TNs.

### Quality assessment and statistics analysis

To rate the caliber of the included studies, we employed an assessment scale based on the Joanna Briggs Institute Tool (**Supplementary Table S1**). Studies were not excluded based on their quality score in order to promote transparency and guarantee that all pertinent evidence in this area was provided. The “Metaprop” module in the R-4.0.2 statistical software package was used for meta-analysis after ensuring consistency (13–17). A 95% confidence interval (95% CI) was generated using Wilson score method, and the DerSimonian–Laird random effects model with Logit transformation was used to compute the pooled prevalence. The heterogeneity among the selected studies was examined using Cochran Q statistics and *I* ^2^ statistics. Estimates having a *p*-value lower than 0.05 for the Q-statistic and *I*² of 50% or greater were deemed to show substantial heterogeneity. Because we anticipated that data from different parts of the world would be used, we employed a random-effects model to pool the prevalence of TNs. A series of leave-one-out diagnostic tests was used for sensitivity analysis, and the results were then verified using a built-in function in “metafor.” As sensitivity analysis failed to uncover outliers in current study, meta-regression was then performed by using a mixed-effects model. In order to determine which possible predictor combination generate the best fit and which predictors are generally the most crucial ones, multivariable meta-regression (multimodel inference) was carried out using the “dmetar” programme. To look at how heterogeneity might be confounding, subgroup analyses were conducted. *P*-value was utilized to evaluate how the groups differed from one another. *P* < 0.05 was deemed to be significant.

## Results

### Literature search results and study characteristics

**Figure 1:**
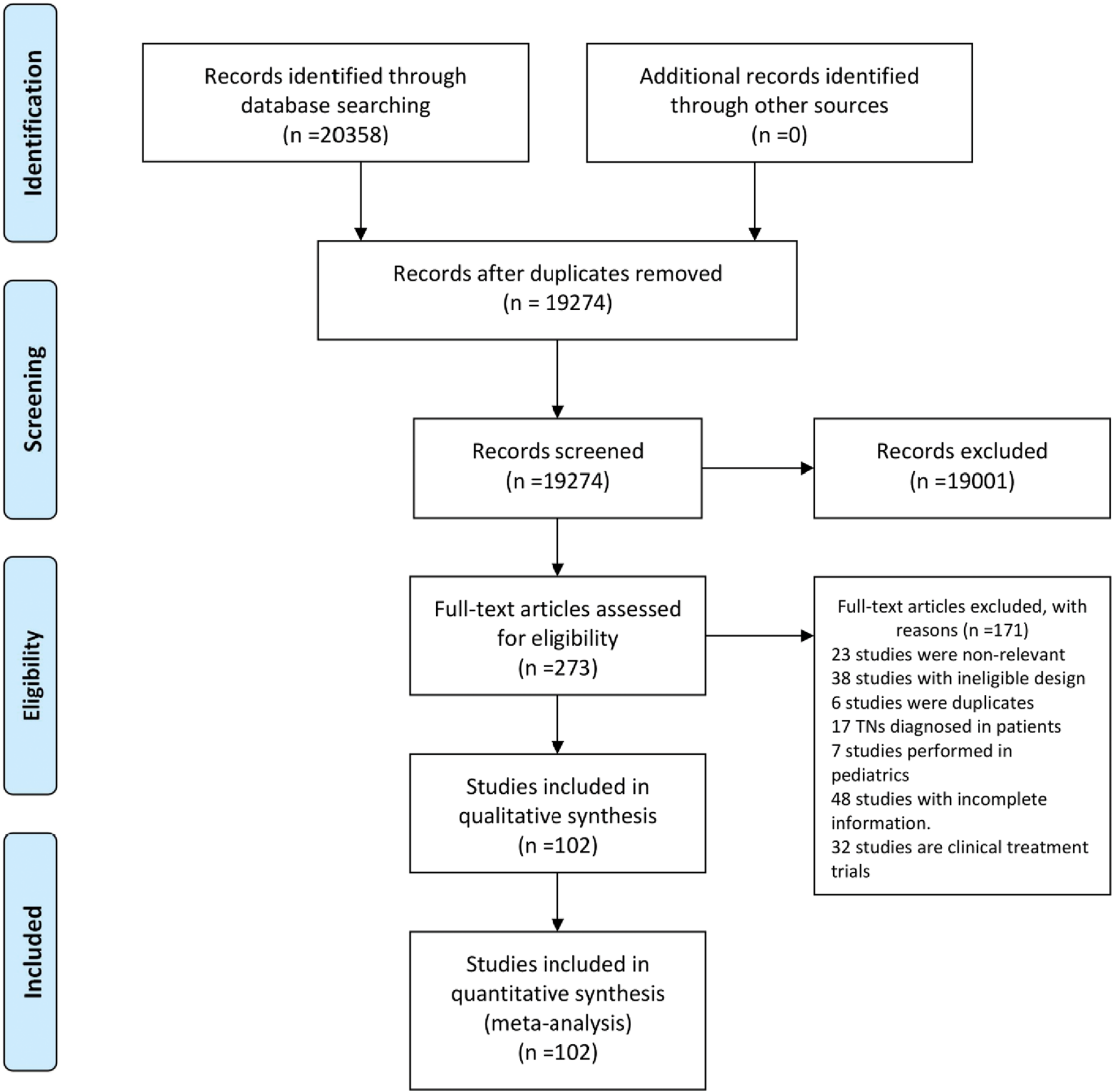
Study selection.

Through a thorough literature search, we identified 20,358 entries. A total of 19,274 records were retained after removing the duplicates. We screened the titles and abstracts and eliminated 19,001 records, which were ineligible. The remaining 273 records’ full texts were assessed for eligibility, of which 171 were excluded. Overall, 102 eligible studies comprising 74,397,483 adults were finally included in the analysis (**Figure 1** and **Supplementary Table S1**). The quality assessment scores for included studies are displayed in the **Supplementary Table S1**.

### Global TNs prevalence among adults in general population

**Figure 2:**
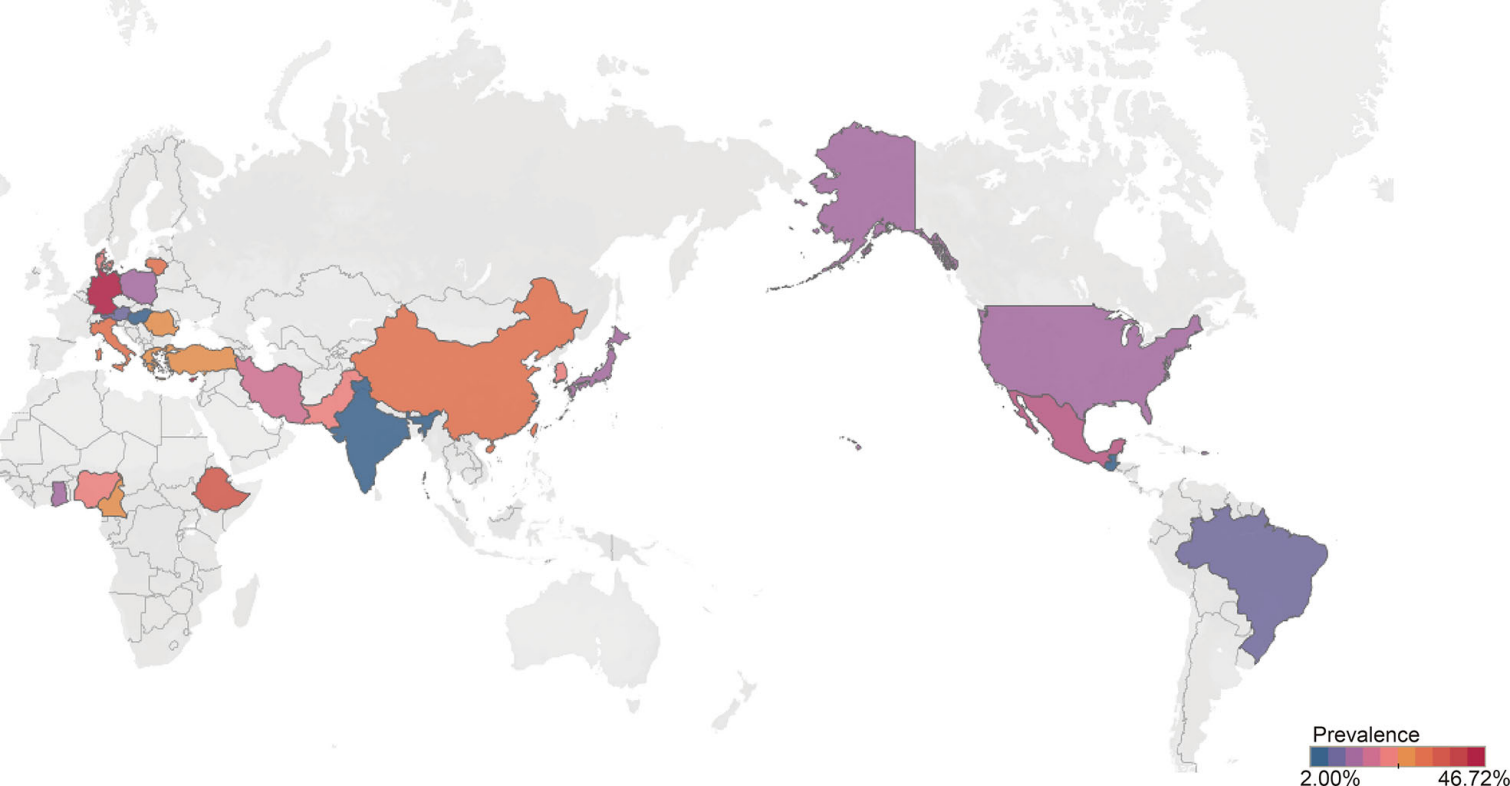
Global prevalence of thyroid nodules among general population.

Based on the TNs definition, we extracted the existing thyroid disorder data to identify individuals with TNs. As a result, a total of 9,276,178 individuals could be identified with TNs from the general population. There was high degree of heterogeneity among the documented results with an overall prevalence rate of 24.83% (95% CI 21.44–28.55), independent of diagnostic techniques (**Figure 2**).

**Figure 3:**
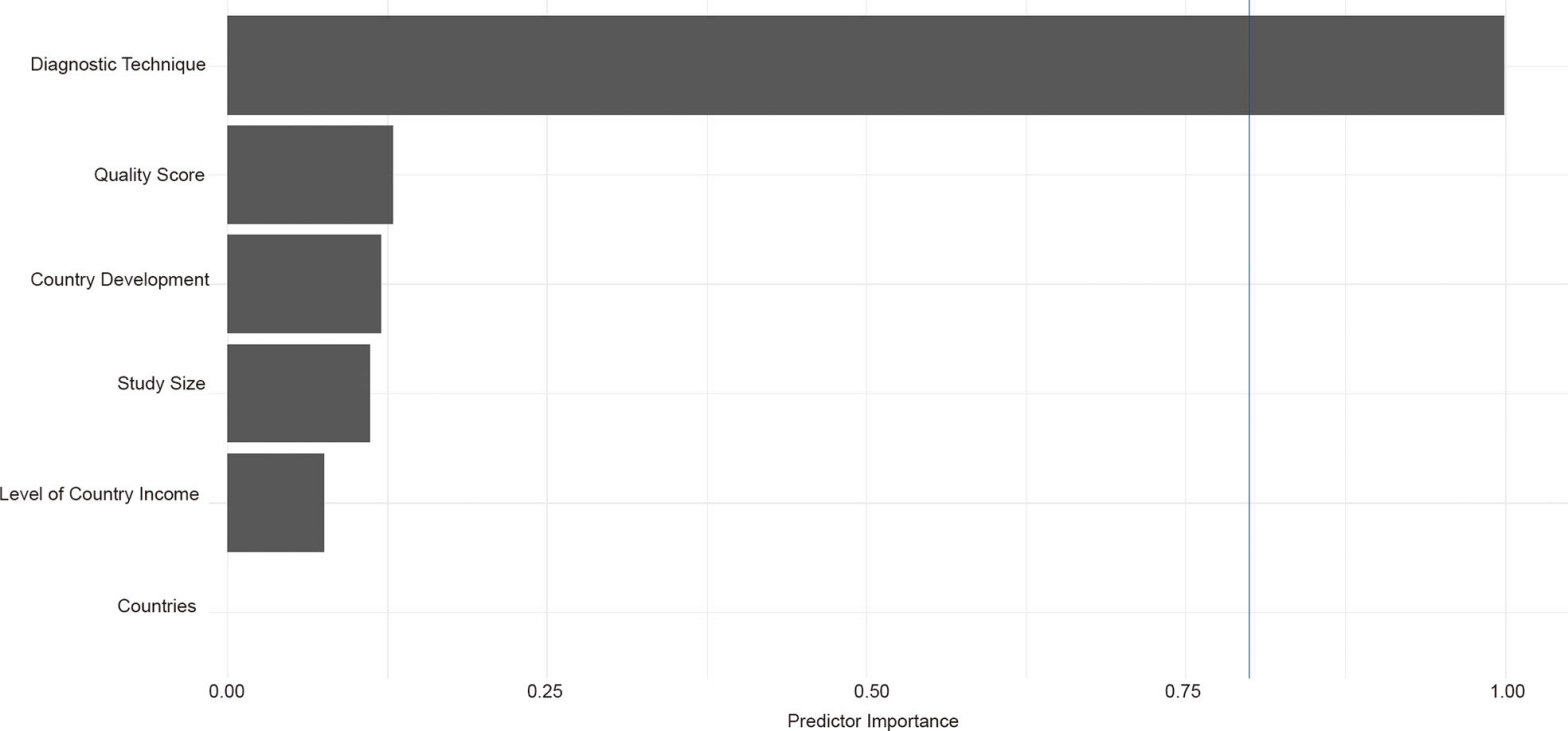
Results of multivariable meta-regression analysis.

In order to further understand the heterogeneity, sensitivity analysis was conducted by performing a set of leave-one-out diagnostic tests (**Supplementary Table S2**) and the results were further verified by using a built-in function in metafor (**Supplementary Figure S1**). Unfortunately, both models failed to identify the outliers. Consequently, meta-regression analysis was performed to further explore the source of heterogeneity. Our univariate meta-regression model indicated that different countries (*R* ^2^ = 0, *p* = 0.29), quality score of study (*R* ^2^ = 0.18, *p* = 0.73), study size (*R* ^2^ = 0.08, *p* = 0.68), development of countries (*R* ^2^ = 0.4, *p* = 0.44), and income of countries (*R* ^2^ = 0.08, *p* = 0.36), were not significantly associated with heterogeneity. The source of heterogeneity across the studies, identified by meta-regression analyses, was the diagnostic techniques (*R* ^2^ = 0.01, *p* < 0.01, **Supplementary Table S3**). By performing multivariable meta-regression, we found the diagnostic techniques with the highest predictor importance of 99.99% (**Figure 3** and **Supplementary Table S4**).

### Subgroup analyses

To confirm the results from meta-regression, subgroup analysis was performed. Thyroid prevalence varied substantially among countries, from 4.75% (Hungary, 95% CI 3.11–7.17) to 46.72% (Cyprus, 95% CI 38.06–55.58, **Table 1**). China holds the most studies for pooled analysis with TN prevalence of 30.23% (95% CI 25.63–35.27). The prevalence of TNs in developing countries (26.36%, 95% CI 22.38–30.76) was slightly higher than that in developed countries (21.57%, 95% CI 15.78–28.75), although without significant difference (*P* = 0.24, **Table 1**). Interestingly, the TN prevalence peaked in upper middle income countries (27.17%, 95% CI 22.99–31.80) but lower in high (21.46%, 95% CI 15.62–28.74) or lower middle countries (19.62%, 95% CI 14.39–26.18, **Table 1**). There is little difference in TN prevalence stratified by study size (*p* = 0.79, **Table 1**). The prevalence of quality assessment score above 8 points (24.67%, 95% CI 21.03–28.72) was slightly lower than that of studies below 8 points without significant difference (25.77%, 95% CI 17.28–36.59, *p* = 0.84, **Table 1**). Little difference was observed when comparing the TNs prevalence stratified by study size (*p* = 0.79). Considering the diagnostic methods, 10 studies used autopsy (6.40%, 95% CI 4.79–8.48) and 92 studies used ultrasound (27.96%, 95% CI 24.50–37.90, *p* < 0.01, **Table 1**). The prevalence of TNs was significantly higher in women (36.51%, 95% CI 31.47–41.87) than in men (23.47%, 95% CI 18.62–29.14, *p* < 0.01, **Table 1**). A total of 16 studies compared the age-stratified TN prevalence. The TN prevalence was 10.59% (95% CI 5.72–18.76), 17.28% (95% CI 11.25–25.63), 24.89% (95% 16.70–35.39), 33.37% (95% CI 22.88–45.82), 41.97% (95% CI 28.63–56.60), and 44.66% (95% CI 18.85–73.71) in age below 30, 30–39, 40–49, 50–59, and 60–69 years and age above 70 years old group, respectively (*p* < 0.01, **Table 1**). Interestingly, an increased prevalence of TNs were observed among overweight (36.96%, 95% CI 30.70–43.69) or obese (40.96%, 95% CI 34.88–47.32) individuals than those with normal weight (30.38%, 95% CI 20.54–42.42, *p* = 0.27, **Table 1**). When compared, the prevalence rates of TNs, there was little difference whether individuals smoked (*p* = 0.87) or drank alcohol (*p* = 0.77, **Supplementary Figures S2, S3**). Moreover, from 2012 to 2022, we found an increased prevalence of TNs (29.29%, 95% CI 24.43–34.67) relative to those diagnosed between 2000 and 2011 (21.53%, 95% CI 17.48–26.21, *p* = 0.02, **Table 1**). Furthermore, we also pooled estimate the characteristics of TNs. The prevalence of solitary TNs (53.03%, 95% CI 46.65–53.91) was marginally higher than that of multiple nodules (46.97%, 95% CI 40.69–53.35, *p* = 0.19, **Supplementary Figure S4**). Notably, we found a pattern of increased prevalence of TNs larger than 1 cm (25.80%, 95%CI 18.56–34.66) than that one below 1 cm (24.58%, 95% CI 20.81–28.78, *p* = 0.79, **Supplementary Figure S5**). Solid TNs (68.39%, 95% CI 25.81–93.08) appears to be most prevalent in TNs, followed by mixed (42.76%, 95% CI 21.96–66.48) and cyst ones (6.27%, 95% CI 3.09–12.30, *p* < 0.01, **Supplementary Figure S6**).

**Table 1:** Subgroup analysis for thyroid nodules prevalence among the general population.

	Studies	TNs	Participants	Prevalence (95% CI)	*P*-value	*I* ^2^
**Overall population**	102	9076178	74397983	24.83 (21.44–28.55)	–	100.00%
**By country**					< 0.01	
South Korea	9	858502	51949155	21.77 (11.16–38.13)		100.00%
Turkey	6	970	3437	24.92 (13.22–41.97)		95.30%
Mainland China	49	8377881	22312395	30.23 (25.63–35.27)		99.60%
Romania	2	102	618	26.68 (3.19–80.05)		99.80%
Italy	5	1025	4661	29.01 (20.20–39.74)		98.80%
Japan	1	924	6422	14.39 (13.55–15.27)		–
Iran	2	156	851	19.10 (13.99–25.52)		76.30%
Ghana	1	36	320	11.25 (8.22–15.20)		–
Germany	4	32723	98142	43.44 (27.07–61.39		99.20%
Cyprus	1	57	122	46.72 (38.06–55.58)		–
Poland	1	20	135	14.81 (9.76–21.85)		–
Cameroon	1	126	446	28.25 (24.27–32.61)		–
India	1	71	4409	1.61 (1.28–2.03)		–
USA	2	52	375	13.82 (10.18–18.50)		25.90%
Pakistan	2	121	519	23.36 (18.66–28.83)		48.60%
Lithuania	1	99	317	31.23 (26.37–36.54)		–
Denmark	2	1959	8588	22.46 (13.08–35.80)		99.50%
Nigeria	1	76	340	22.35 (18.24–27.09)		–
Greece	2	180	462	24.43 (2.16–82.55)		99.10%
Austria	1	10	118	8.47 (4.62–15.04)		–
Mexico	2	483	2701	9.36 (1.80–36.85)		97.20%
Brazil	1	13	166	7.83 (4.60–13.02)		–
Ethiopia	1	97	290	33.45 (28.25–39.08)		–
Hungary	2	21	443	4.75 (3.11–7.17)		0.00%
Guatemala	1	3	150	2.00 (0.65–6.02)		–
**By sex**					<0.01	
Male	42	3651461	63249110	23.47 (18.62–29.14)		100.00%
Female	49	5464843	36307625	36.51 (31.47–41.87)		100.00%
**By age**					0.5	
<40	3	681	1522	41.3 (30.3–53.3)		94.8%
40-60	3	911	1564	56.3 (26.3–82.3)		99.2%
>60	4	936	1582	51.8 (32.7–70.3)		97.9%
**By development**					0.24	
Developed	32	863804	51973102	21.57 (15.78–28.75)		99.90%
Developing	70	8412374	22424881	26.36 (22.38–30.76)		99.20%
**By income**					0.11	
High	33	895740	52073639	21.46 (15.62–28.74)		99.30%
Upper-middle	64	8380044	22322387	27.17 (22.99–31.80)		99.50%
Lower-middle	5	394	1957	19.62 (14.39–26.18)		89.80%
**By study period**					0.02	
2000-2011	46	109401	343684	21.53 (17.48–26.21)		99.50%
2012-2022	53	8322465	21909748	29.29 (24.43–34.67)		99.90%
**By study quality score**					0.84	
<8	14	5595944	14500765	25.77 (17.28–36.59)		99.70%
≥8	88	3680234	59897218	24.67(21.03–28.72)		100.00%
**By study size**					0.79	
<10000	82	60876	199331	24.58 (20.81–28.78)		99.50%
≥10000	20	9215302	74198652	25.80 (18.56–34.66)		100.00%
**By diagnostic techniques**					<0.01	
Autopsy	10	143	2121	6.40 (4.79–8.48)		63.30%
Ultrasound	92	1715252	2266104	27.96 (24.50–31.70)		100.00%
**By body mass index**					0.27	
Normal weight	9	2398375	6884626	30.38 (20.54–42.42)		99.90%
Overweight	10	2008124	4847149	36.96 (30.70–43.69)		99.20%
obese	53	917443	1901969	40.96 (34.88–47.32)		100.00%

## Discussion

The TN prevalence in iodine-sufficient populations is around 5%, depending on age and sex, according to physical examination (18). Clinicians, on the other hand, encountered a substantially greater percentage of patients with undetected TNs, which is partly owing to the increased use of diagnostic imaging for purposes unrelated to the thyroid, which has resulted in the detection of asymptomatic nodules (19). In this study, we estimated the global prevalence of TNs in the general population through repurposing and redefining existing epidemiological data on thyroid disorders. TNs were found in one of every four people in the general population, according to our findings. Alarmingly, TNs have become a pandemic irrespective of country development and economic status.

Iodine is required for the production of thyroid hormones, particularly that of thyroxine and triiodothyronine. Thyroid-hormone abnormalities can be caused by both insufficient and excessive iodine consumption, and the appearance of goiter and TNs indicates thyroid disease (20). Iodine can be added to salt to treat iodine-deficient diets and, consequently, many countries have salt iodination programs to prevent iodine deficiency in their populations. When a country starts such a program, the rates of hypothyroidism decrease significantly. However, what is less well known is whether there is any change in the rate of TNs after starting a salt iodination program. A previous study evaluated the frequency of goiter and TNs in mainland China before and after the universal salt iodization program (21). To our surprise, in 2002, when the universal salt iodization program was launched, the prevalence of TNs rose considerably, implying the possible risk of excessive iodine consumption (22). However, so far, there is no exact mechanism regarding excessive iodine leading to TNs (23, 24). Because of the diverse epidemiological conditions across the world, there has been a long-standing debate over the association between the prevalence of TNs and iodine diet. The rate of TNs ranged from 2.6% (25) in iodine-adequate countries to the iodine-deficient area revealed a prevalence of 20.2% (26). Greater perplexing, Mexico, which was formerly somewhat iodine deficient, now consumes more than average iodine consumption, and the prevalence of locally detected TNs was 19.6% (27). According to latest evidence, non-iodized salt may not arise the risk of TNs (28). In current study, our pooled estimates indicated a dramatically increased prevalence of TNs from 2012 to 2022 when compared with 2000–2011. On the one hand, because the majority of the nations included had a regular iodized program, we cannot rule out the possibility that this was caused by an excessive consumption of iodine salt. On the other hand, with the fast improvement of US technology in recent years and the availability of high-frequency transducers, tiny thyroid lesions may now be detected.

The unbalanced prevalence of TNs among general population has been observed stratified by different diagnostic techniques. Ultrasound has been used to describe detection and characterization of TNs for more than 5 decades (29). Since then, several studies have been conducted in order to determine the validity of thyroid US in the diagnosis of TNs. The sensitivity and specificity of TNs, according to the current report, ranged from 70.6 to 97.4% and 29.3 to 90.4%, respectively (30). The use of autopsy in the identification of TNs was not frequent. A recent study included 35 papers comprising 12,834 autopsies; the pooled estimate prevalence of differentiated thyroid cancer (DTC) was 11.2% (31). To our surprise, our pooled estimates of TNs diagnosed by autopsy were even lower than that of DTC. This could be partially explained by most papers involved in DTC study published before 1990s but the worldwide iodized salt program started 10 years after that. Furthermore, when compared with ultrasound, we discovered a lower prevalence of TNs detected by autopsy. This is partly owing to ultrasound’s excellent sensitivity, which can detect TNs as small as 2 mm. In addition, the findings from autopsy may have some limitations, principally the unknown validity of pathologic assessment at autopsy. For example, although not reported in these studies, thyroid glands may show autolysis on histologic examination, and tiny lesions may have the potential to autolyze and might falsely decrease estimates of TN prevalence. Another explanation was missing detail of methods; because many studies were old, contact with authors for details of how to identify the TNs from the autopsy was not possible.

Although there is a lack of evidence that TNs have a strong etiological association with iodine nutrition, the prevalence of TNs is affected by sex, age, body mass index, and head-and-neck radiation exposure history (32, 33). A common characteristic in the prevalence of thyroid disorders is in female preponderance. Based on our present results, women had a 1.5-fold greater TN prevalence than men, indicating a lower female-to-male rate ratio than earlier findings (3–4: 1) (34). Estrogen (estrogen receptors are found in thyroid follicular cells in normal and neoplastic tissue), which also affects thyroid-stimulating hormone and may have a role in the production of TNs, has a big impact on this (35). Estrogen-induced increases in thyroid follicular cell proliferation have been shown *in vitro*, suggesting that excessive estrogen levels may be a risk factor for goiter and TNs (36). Furthermore, the prevalence of TNs has been shown to be about four times higher in those over the age of 70 years than in people under the age of 30 years. Our findings add to our understanding of thyroid nodular disease in adults by demonstrating that becoming older significantly increases the probability of TN development. In this meta-analysis, we found that obesity was correlated with prevalence of TNs, which highlights the important role of metabolic disturbances in the formation of TNs. Impaired metabolism has recently been described as an independent risk factor for increased thyroid volume and nodule prevalence in multiple earlier investigations (37–39). Thyroid nodules and thyroid cancer are more common in obese people (40, 41). A thyroid-stimulating hormone (TSH)–dependent mechanism involving leptin signaling could account for the link between TNs and metabolic disturbances. Thyrotropin-releasing hormone (TRH) is released more frequently as a result of leptin, both directly and indirectly increasing TRH production (42). Serum leptin concentration increases in proportion to increasing fat mass, and insulin administration increases serum leptin levels (43). Thus, it has been hypothesized that obesity and insulin resistance cause TSH secretion to rise as a result of leptin signaling, which causes thyroid volume expansion and nodule formation.

A unique strength in this study is that we have comprehensively estimate TN prevalence by retrospectively repurposing existing data on thyroid disorders. Nevertheless, there are some limitations in the current study. First, limited data, in particular from Oceania, challenged the accuracy in estimation. Second, we observed a high heterogeneity in our pooled estimations, as all the included studies were cross-sectional studies. Although we performed meta-regression and subgroup analyses, additional covariates such as race, level of urine iodine, and amount of iodine intake cannot be investigated due to the scarcity of available data.

In conclusion, TNs have an astonishingly high prevalence rate in the general populations. This calls for attention and dedicated action from primary care physicians, specialists, health policy makers, and the general public alike.

## Data availability statement

The original contributions presented in the study are included in the article/**Supplementary Material**. Further inquiries can be directed to the corresponding authors.

## Author contributions

CM, YT and XM performed the study, acquisition and analysis of data. YoL performed literature database searching. YoL, YT and ZL discussed the data; conceive the idea and revising the manuscript. YN and MY provided technical assistance. YT drafted the manuscript. All authors have read the manuscript and provided critical feedback. CM, YT and XM contributed equally and share co-first authorship. All authors contributed to the article and approved the submitted version.

## Funding

This research is supported by the fellowship of China Postdoctoral Science Foundation (2021M702340), Sichuan Science and Technology Program (2021ZYCD016, 2020YFS0573, 2022NSFSC1441), Key Research and Development Program of Science and Technology Department of Sichuan Province 2019YFS0360.

## Acknowledgments

We thanked Ningning Chao, Shunqiang Mao, Zhiqiang Liu, Ying Yang from Department of Respiratory and Critical Care Medicine, National Clinical Research Center for Geriatrics, Center of Precision Medicine, Precision Medicine Key Laboratory of Sichuan Province, Frontiers Science Center for Disease-related Molecular Network, West China Hospital, West China School of Medicine, Sichuan University, Chengdu, Sichuan, China for providing technique assistance.

## Conflict of interest

The authors declare that the research was conducted in the absence of any commercial or financial relationships that could be construed as a potential conflict of interest.

## Publisher’s note

All claims expressed in this article are solely those of the authors and do not necessarily represent those of their affiliated organizations, or those of the publisher, the editors and the reviewers. Any product that may be evaluated in this article, or claim that may be made by its manufacturer, is not guaranteed or endorsed by the publisher.
