# Supplementary material for: Mapping global epidemiology of thyroid nodules among general population: A systematic review and meta-analysis
Source: Front Oncol. 2022 Nov 10;12:1029926. doi: 10.3389/fonc.2022.1029926 (PMC9685339; doi:10.3389/fonc.2022.1029926)

***Supplementary File to***

**Mapping global epidemiology of thyroid nodules among general population: a systematic review and meta-analysis**

Xin Ming, Ye Tian, Yang Liu, Menglin Yao, Yinyun Ni, Yong Liu, Zhihui Li

**Supplementary Methods 1. Searching strategy for prevalence of thyroid nodules among the general populations.**

| embase.com | 10587 | 9987 |
| --- | --- | --- |
| Medline Ovid | 6672 | 6473 |
| Web of science Core Collection | 2880 | 2778 |
| Cochrane CENTRAL | 219 | 36 |
| **Overall** | **20358** | **19274** |

**embase.com**

('thyroid nodules'/ AND ('epidemiological data'/de OR 'epidemiology'/de OR 'geographic distribution' /exp OR 'patient volume'/de OR prevalence/exp OR geography/de OR 'geographic names'/exp OR (epidemiolog* OR ((geograph* OR global*) NEAR/3 (distribut*)) OR incidenc* OR (patient* NEAR/3 volume*) OR prevalen*):ab,ti) NOT ([animals]/lim NOT [humans]/lim) NOT ('case report'/de OR 'case report*':ti) NOT ([Conference Abstract]/lim)

**Medline Ovid**

(Thyroid nodules/ AND (Epidemiological Monitoring/ OR Epidemiology/ OR Epidemiology.fs. OR exp Incidence/ OR exp Prevalence/ OR Geography/ OR exp Geographic Locations/ OR (epidemiolog* OR ((geograph* OR global*) ADJ3 (distribut*)) OR incidenc* OR (patient* ADJ3 volume*) OR prevalen*).ab,ti.) NOT (exp animals/ NOT humans/) NOT (case report/ OR case report*.ti.) NOT (news OR congres* OR abstract* OR book* OR chapter* OR dissertation abstract*)..

**Web of science Core Collection**

TS=(thyroid nodules*) AND ((epidemiolog* OR ((geograph* OR global*) NEAR/2 (distribut*)) OR incidenc* OR (patient* NEAR/2 volume*) OR prevalen*)) NOT ((animal* OR rat OR rats OR mouse OR mice OR murine OR dog OR dogs OR canine OR cat OR cats OR feline OR rabbit OR cow OR cows OR bovine OR rodent* OR sheep OR ovine OR pig OR swine OR porcine OR veterinar* OR chick* OR zebrafish* OR baboon* OR nonhuman* OR primate* OR cattle* OR goose OR geese OR duck OR macaque* OR avian* OR bird* OR fish*) NOT (human* OR patient* OR women OR woman OR men OR man))) NOT TI=( "case report*") AND L DT=(article)

**Cochrane CENTRAL**

(thyroid nodules*) AND ((epidemiolog* OR ((geograph* OR global*) NEAR/3 (distribut*)) OR incidenc* OR (patient* NEAR/3 volume*) OR prevalen*):ab,ti)

**Supplementary Table 1. Characteristics of included studies.**

| Author | Year | Period | Country | Income | Develop | Quality | Diagnostic | Cases | Total | Men | Woman | Age<30 | Age 30-39 | Age 40-49 | Age 50-59 | Age 60-69 | Age >70 |
| --- | --- | --- | --- | --- | --- | --- | --- | --- | --- | --- | --- | --- | --- | --- | --- | --- | --- |
| Moon et al | 2018 | 2004-2010 | Korea | High | Developed | 8 | US | 24757 | 72319 | 9919/36710 | 14838/35609 | 250/1935 | 2378/11411 | 6933/23649 | 8655/22113 | 5197/10763 | 1344/2448 |
| Shin et al | 2016 | 2009-2010 | Korea | High | Developed | 8 | US | 764 | 1990 | 223/691 | 541/1299 |  |  |  |  |  |  |
| Wang et al | 2019 | 2015-2017 | Mainland China | Upper-Middle | Developing | 9 | US | 2991 | 6070 |  |  |  |  |  |  |  |  |
| Morna et al | 2022 | 2019 | Ghana | Lower-Middle | Developing | 8 | US | 36 | 320 | 5/125 | 31/191 | 2/70 | 5/63 | 6/61 | 8/57 | 15/69 |  |
| Moifo et al | 2017 | 2015 | Cameroon | Lower-Middle | Developing | 8 | US | 126 | 446 |  |  |  |  |  |  |  |  |
| Bello et al | 2013 | 2013 | Nigeria | Lower-Middle | Developing | 8 | US | 76 | 340 |  |  |  |  |  |  |  |  |
| Karaszewski et al | 2006 | 2005 | Poland | High | Developed | 8 | US | 20 | 135 |  |  |  |  |  |  |  |  |
| Khan et al | 2021 | 2019 | Pakistan | Upper-Middle | Developing | 9 | US | 65 | 250 |  |  | 18/137 | 16/55 | 12/27 | 11/16 | 8/15 |  |
| Tadesse et al | 2020 | 2015 | Ethiopia | High | Developed | 6 | US | 97 | 290 |  |  |  |  |  |  |  |  |
| Sharon et al | 2014 | 2009-2011 | Mainland China | Upper-Middle | Developing | 8 | US | 8480 | 19895 | 4661/11678 | 3819/8217 |  |  |  |  |  |  |
| Gnarini et al | 2013 | 2011 | Italy | High | Developed | 8 | US | 91 | 201 | 26/66 | 73/135 |  |  |  |  |  |  |
| Armanini et al | 2003 | 2003 | Italy | High | Developed | 8 | US | 17 | 80 |  |  |  |  |  |  |  |  |
| Liebeskind et al | 2005 | 2003 | USA | High | Developed | 8 | US | 35 | 225 |  |  |  |  |  |  |  |  |
| Jiang et al | 2016 | 2013 | Mainland China | Upper-Middle | Developing | 8 | US | 3100 | 6324 | 967/3100 | 2133/3100 |  |  |  |  |  |  |
| Akarsu et al | 2005 | 2005 | Turkey | Upper-Middle | Developing | 8 | US | 94 | 340 |  |  |  |  |  |  |  |  |
| Kocak et al | 2014 | 2006-2008 | Turkey | Upper-Middle | Developing | 9 | US | 662 | 2500 | 301/1230 | 361/1270 | 87/694 | 138/552 | 157/505 | 124/341 | 85/223 | 71/185 |
| Wang et al | 2015 | 2011 | Mainland China | Upper-Middle | Developing | 8 | US | 1965 | 6503 |  | 1965/6503 |  |  |  |  |  |  |
| Song et al | 2018 | 2009-2010 | Mainland China | Upper-Middle | Developing | 8 | US | 187 | 1482 | 77/187 | 110/187 |  |  |  |  |  |  |
| Yang et al | 2019 | 2017-2019 | Mainland China | Upper-Middle | Developing | 8 | US | 2074 | 3534 | 1310/2387 | 764/1047 |  |  |  |  |  |  |
| Chen et al | 2021 | 2011-2012 | Mainland China | Upper-Middle | Developing | 9 | US | 238 | 1279 | 78/524 | 160/755 |  |  |  |  |  |  |
| Xu et al | 2021 | 2015-2020 | Mainland China | Upper-Middle | Developing | 7 | US | 41547 | 121702 | 21813/73139 | 19734/48563 | 4912/25455 | 8527/35672 | 10080/27063 | 9845/20062 | 8183/13450 |  |
| Tian et al | 2020 | 2017-2018 | Mainland China | Upper-Middle | Developing | 8 | US | 1350 | 3661 |  |  |  |  |  |  |  |  |
| Song et al | 2016 | 2009 | Mainland China | Upper-Middle | Developing | 8 | US | 1428 | 5144 | 561/2597 | 867/2547 | 109/613 | 164/706 | 327/1082 | 441/1334 | 268/656 |  |
| Sun et al | 2020 | 2011-2012 | Mainland China | Upper-Middle | Developing | 8 | US | 2032 | 12698 | 756/5506 | 1276/7192 |  |  |  |  |  |  |
| Fan et al | 2018 | 2015 | Mainland China | Upper-Middle | Developing | 8 | US | 707 | 2647 |  |  |  |  |  |  |  |  |
| Chen et al | 2013 | 2010 | Mainland China | Upper-Middle | Developing | 9 | US | 2822 | 9412 | 1014/4202 | 1808/5210 |  |  |  |  |  |  |
| Guo et al | 2014 | 2011 | Mainland China | Upper-Middle | Developing | 8 | US | 4439 | 9533 | 1341/3375 | 3098/6158 |  |  |  |  |  |  |
| Kamran et al | 2014 | 2012-2013 | Pakistan | Upper-Middle | Developing | 8 | US | 56 | 269 | 21/129 | 35/140 | 4/68 | 15/75 | 12/44 | 12/47 | 13/35 |  |
| Yao et al | 2018 | 2018 | Mainland China | Upper-Middle | Developing | 9 | US | 679 | 874 |  |  |  |  |  |  |  | 679/874 |
| Batolotta et al | 2006 | 2006 | Italy | High | Developed | 8 | US | 233 | 704 |  |  |  |  |  |  |  |  |
| Acar et al | 2014 | 2009-2012 | Turkey | Upper-Middle | Developing | 8 | US | 161 | 315 | 93/190 | 68/125 |  |  |  |  |  |  |
| Zhu et al | 2012 | 2010 | Mainland China | Upper-Middle | Developing | 8 | US | 3514 | 9612 | 2283/7299 | 1231/2313 |  |  |  |  |  |  |
| Zeng et al | 2022 | 2021 | Mainland China | Upper-Middle | Developing | 8 | US | 543 | 1048 |  |  |  |  |  |  |  |  |
| Zhu et al | 2021 | 2018 | Mainland China | Upper-Middle | Developing | 6 | US | 3658551 | 9381032 | 1533301/4860891 | 2125250/4520141 | 388393/1660869 | 767085/2708480 | 867053/2136789 | 922811/1775293 | 530886/841324 | 182323/258277 |
| Li et al | 2021 | 2017 | Mainland China | Upper-Middle | Developing | 8 | US | 2578231 | 6985956 | 1101643/3681362 | 1476513/3304574 |  |  |  |  |  |  |
| Xu et al | 2021 | 2010 | Mainland China | Upper-Middle | Developing | 8 | US | 2228 | 6793 | 646/2516 | 1582/4277 |  |  |  |  |  |  |
| Liu et al | 2013 | 2013 | Mainland China | Upper-Middle | Developing | 8 | US | 224 | 1205 | 75/531 | 149/674 |  |  |  |  |  |  |
| Wang et al | 2021 | 2017-2019 | Mainland China | Upper-Middle | Developing | 9 | US | 20784 | 51637 | 10383/29842 | 10401/21795 |  |  |  |  |  |  |
| Lai et al | 2020 | 2010-2020 | Mainland China | Upper-Middle | Developing | 8 | US | 23868 | 309576 |  |  |  |  |  |  |  |  |
| Kim et al | 2016 | 2011-2013 | Korea | High | Developed | 7 | US | 1338 | 3655 |  | 1338/3655 |  |  |  |  |  |  |
| Guo et al | 2019 | 2015 | Mainland China | Upper-Middle | Developing | 8 | US | 681 | 2606 | 315/1338 | 366/1268 |  |  |  |  |  |  |
| Zou et al | 2019 | 2014-2018 | Mainland China | Upper-Middle | Developing | 8 | US | 2961 | 9146 | 1593/6119 | 1368/3027 | 487/2441 | 1179/3792 | 914/2227 | 318/599 | 63/87 |  |
| Feng et al | 2017 | 2014 | Mainland China | Upper-Middle | Developing | 9 | US | 1150 | 6494 | 312/2427 | 838/4067 |  |  |  |  |  |  |
| Karger et al | 2009 | 2009 | Germany | High | Developed | 7 | US | 192 | 424 |  |  |  |  |  |  |  |  |
| Shokri et al | 2020 | 2020 | Iran | Lower-Middle | Developing | 8 | US | 97 | 588 |  | 97/588 |  |  |  |  |  |  |
| Gao et al | 2019 | 2016-2017 | Mainland China | Upper-Middle | Developing | 8 | US | 314 | 2353 |  | 314/2353 |  |  |  |  |  |  |
| Piciu et al | 2015 | 2014-2015 | Cyprus | High | Developed | 7 | US | 57 | 122 |  | 57/122 |  |  |  |  |  |  |
| Piciu et al | 2015 | 2014-2015 | Romania | Upper-Middle | Developing | 9 | US | 51 | 92 |  | 51/92 |  |  |  |  |  |  |
| Meng et al | 2022 | 2016-2017 | Mainland China | Upper-Middle | Developing | 8 | US | 354 | 2488 |  |  |  |  |  |  |  |  |
| Solares et al | 2005 | 2000 | USA | High | Developed | 8 | Autopsy | 17 | 150 |  |  |  |  |  |  |  |  |
| Zhang et al | 2021 | 2017 | Mainland China | Upper-Middle | Developing | 7 | US | 1869742 | 4920536 | 803102/2598909 | 1066640/2321627 |  |  |  |  |  |  |
| Rizza et al | 2020 | 2016 | Italy | High | Developed | 8 | US | 96 | 299 |  |  |  |  |  |  |  |  |
| Marwaha et al | 2012 | 2012 | India | High | Developing | 8 | US | 71 | 4409 |  |  |  |  |  |  |  |  |
| Karatoprak et al | 2012 | 2011 | Turkey | Upper-Middle | Developing | 8 | US | 24 | 91 |  |  |  |  |  |  |  |  |
| Li et al | 2020 | 2020 | Mainland China | Upper-Middle | Developing | 9 | US | 16164 | 78470 |  |  |  |  |  |  |  |  |
| Li et al | 2007 | 2007 | Mainland China | Upper-Middle | Developing | 7 | US | 166 | 3813 |  |  |  |  |  |  |  |  |
| Li et al | 2010 | 2010 | Mainland China | Upper-Middle | Developing | 8 | US | 1921 | 15008 |  |  |  |  |  |  |  |  |
| Shan et al | 2016 | 2016 | Mainland China | Upper-Middle | Developing | 8 | US | 1925 | 15008 |  |  |  |  |  |  |  |  |
| Guth et al | 2009 | 2006-2007 | Germany | High | Developed | 8 | US | 432 | 635 |  |  |  |  |  |  |  |  |
| Dauksiene et al | 2017 | 2012 | Lithuania | High | Developed | 7 | US | 99 | 317 | 28/142 | 71/175 |  |  |  |  |  |  |
| Zheng et al | 2015 | 2013 | Mainland China | Upper-Middle | Developing | 8 | US | 1618 | 3084 |  | 1618/3084 | 23/81 | 117/338 | 337/758 | 571/1026 | 467/739 | 103/147 |
| Knudsen et al | 2002 | 2000 | Denmark | High | Developed | 9 | US | 759 | 4421 |  |  |  |  |  |  |  |  |
| Li et al | 2020 | 2014-2018 | Mainland China | Upper-Middle | Developing | 8 | US | 986 | 2608 | 519/1887 | 467/721 |  |  |  |  |  |  |
| Suk et al | 2006 | 2002-2003 | Korea | High | Developed | 8 | US | 3040 | 7440 | 370/1272 | 2602/6108 |  |  |  |  |  |  |
| Panagiotou et al | 2017 | 2017 | Greece | High | Developed | 8 | US | 168 | 302 |  |  |  |  |  |  |  |  |
| Liu et al | 2020 | 2015-2019 | Mainland China | Upper-Middle | Developing | 8 | US | 64466 | 133698 |  |  |  |  |  |  |  |  |
| Wang et al | 2021 | 2017-2019 | Mainland China | Upper-Middle | Developing | 9 | US | 13036 | 43411 | 6411/25315 | 6625/18096 |  |  |  |  |  |  |
| Shen et al | 2013 | 2012 | Mainland China | Upper-Middle | Developing | 8 | US | 435 | 988 | 240/621 | 195/367 |  |  |  |  |  |  |
| Lopez et al | 2011 | 2005-2007 | Mexico | Upper-Middle | Developing | 8 | US | 471 | 2401 |  |  |  |  |  |  |  |  |
| Reiner et al | 2004 | 2001-2002 | Germany | High | Developing | 8 | US | 31868 | 96278 |  |  |  |  |  |  |  |  |
| Yin et al | 2014 | 2009 | Mainland China | Upper-Middle | Developing | 8 | US | 4728 | 13522 |  |  |  |  |  |  |  |  |
| Imani et al | 2010 | 2006 | Iran | Lower-Middle | Developing | 7 | US | 59 | 263 |  |  | 9/59 | 6/59 | 24/93 | 20/50 |  |  |
| Liu et al | 2017 | 2012-2014 | Mainland China | Upper-Middle | Developing | 7 | US | 24010 | 67781 | 10129/33020 | 13881/34761 |  |  |  |  |  |  |
| Ding et al | 2017 | 2014-2015 | Mainland China | Upper-Middle | Developing | 8 | US | 2201 | 6798 | 854/3289 | 1352/3509 |  |  |  |  |  |  |
| Oh et al | 2010 | 2003-2005 | Korea | High | Developed | 8 | US | 6023 | 15415 | 740/1951 | 5283/13464 |  | 367/1484 | 2031/6148 | 2245/5278 | 1380/2505 |  |
| Kim et al | 2008 | 2005-2008 | Korea | High | Developed | 9 | US | 1082 | 4832 | 396/2405 | 686/2427 | 4/56 | 94/749 | 274/1596 | 371/1412 | 271/839 | 68/180 |
| Zou et al | 2020 | 2018-2019 | Mainland China | Upper-Middle | Developing | 8 | US | 863 | 2309 | 501/1503 | 362/806 |  |  |  |  |  |  |
| Kim et al | 2012 | 2009 | Korea | High | Developed | 8 | US | 1063 | 7763 | 751/4681 | 852/3082 |  |  |  |  |  |  |
| Delitala et al | 2015 | 2001 | Italy | High | Developed | 8 | US | 588 | 3377 |  |  |  |  |  |  |  |  |
| Du et al | 2014 | 2014 | Mainland China | Upper-Middle | Developing | 8 | US | 329 | 2147 |  |  |  |  |  |  |  |  |
| Wang et al | 2014 | 2011 | Mainland China | Upper-Middle | Developing | 8 | US | 2886 | 10050 |  |  |  |  |  |  |  |  |
| Yan et al | 2015 | 2011 | Mainland China | Upper-Middle | Developing | 9 | US | 229 | 1334 |  |  |  |  |  |  |  |  |
| Gao et al | 2020 | 2016-2017 | Mainland China | Upper-Middle | Developing | 8 | US | 284 | 2163 |  |  |  |  |  |  |  |  |
| Sahin et al | 2014 | 2012 | Turkey | Upper-Middle | Developing | 7 | US | 25 | 83 |  | 25/83 |  |  |  |  |  |  |
| Kim et al | 2007 | 2005 | Korea | High | Developed | 8 | US | 152 | 1081 | 152/1081 |  |  | 12/117 | 74/581 | 65/380 | 1/3 |  |
| Kwon et al | 2018 | 2006-2015 | Korea | High | Developed | 8 | US | 820283 | 51834660 | 133515/51834660 | 692743/25901411 | 31148/6881546 | 91382/7824337 | 169997/8871241 | 248945/8332932 | 173674/5064740 | 76976/3153746 |
| Li et al | 2020 | 2017-2018 | Mainland China | Upper-Middle | Developing | 8 | US | 504 | 853 |  |  |  |  |  |  |  |  |
| Wang et al | 2019 | 2015-2017 | Mainland China | Upper-Middle | Developing | 8 | US | 2991 | 6070 |  |  |  |  |  |  |  |  |
| Pan et al | 2020 | 2017 | Mainland China | Upper-Middle | Developing | 8 | US | 955 | 2040 |  |  |  |  |  |  |  |  |
| Krejbjerg et al | 2014 | 2008-2010 | Denmark | High | Developed | 8 | US | 1200 | 4167 |  |  |  |  |  |  |  |  |
| Brauer et al | 2005 | 2002 | Germany | High | Developed | 9 | US | 231 | 805 | 30/156 | 201/649 |  |  |  |  |  |  |
| Neuhold et al | 2001 | 2001 | Austria | High | Developed | 8 | Autopsy | 10 | 118 | 6/57 | 4/61 |  |  |  |  |  |  |
| de matos et al | 2006 | 2000 | Brazil | Upper-Middle | Developing | 8 | Autopsy | 13 | 166 |  |  |  |  |  |  |  |  |
| Kovacs et al | 2005 | 2005 | Hungary | High | Developed | 8 | Autopsy | 11 | 222 |  |  |  |  |  |  |  |  |
| Szolnok et al | 2005 | 2005 | Hungary | High | Developed | 7 | Autopsy | 10 | 221 |  |  |  |  |  |  |  |  |
| Mitselou et al | 2002 | 2000 | Greece | High | Developed | 8 | Autopsy | 12 | 160 |  |  |  |  |  |  |  |  |
| Solares et al | 2005 | 2000 | Guatemala | Upper-Middle | Developed | 8 | Autopsy | 3 | 150 |  |  |  |  |  |  |  |  |
| Tanriover et al | 2011 | 2007 | Turkey | Upper-Middle | Developing | 8 | Autopsy | 4 | 108 |  |  |  |  |  |  |  |  |
| Mohorea et al | 2021 | 2017-2020 | Romania | Upper-Middle | Developing | 7 | Autopsy | 51 | 526 |  |  |  |  |  |  |  |  |
| Ramirez et al | 2017 | 2017 | Mexico | Upper-Middle | Developing | 8 | Autopsy | 12 | 300 |  |  |  |  |  |  |  |  |
| Hurtado et al | 2011 | 2011 | Mexcio | Upper-Middle | Developing | 8 | US | 471 | 2401 |  |  |  |  |  |  |  |  |
| Kikuchi et al | 2013 | 2013 | Japan | High | Developed | 8 | US | 924 | 6422 |  |  |  |  |  |  |  |  |

| Author | Size <1cm | Size >1cm | Single | Multi | Solid | Cyst | Mixed | Normal | Overweight | Obese | Alcohol (Yes) | No | Smoke(Yes) | No |
| --- | --- | --- | --- | --- | --- | --- | --- | --- | --- | --- | --- | --- | --- | --- |
| Moon et al |  |  |  |  |  |  |  |  |  |  |  |  |  |  |
| Shin et al | 578/764 | 186/764 | 467/764 | 297/764 |  |  |  |  |  |  |  |  |  |  |
| Wang et al | 2593/2991 | 398/2991 | 1479/2991 | 1512/2991 |  |  |  |  |  |  |  |  |  |  |
| Morna et al | 20/36 | 28/36 | 23/36 | 13/36 | 34/36 | 2/36 |  | 17/228 | 8/58 | 11/34 | 2/68 | 23/219 |  |  |
| Moifo et al |  |  |  |  |  |  |  |  |  |  |  |  |  |  |
| Bello et al |  |  |  |  |  |  |  |  |  |  |  |  |  |  |
| Karaszewski?et al |  |  | 18/20 | 2/20 |  |  |  |  |  |  |  |  |  |  |
| Khan et al |  |  | 21/65 | 44/65 | 33/65 | 4/65 | 21/65 |  |  |  |  |  |  |  |
| Tadesse et al | 72/97 | 25/97 | 61/97 | 36/97 |  |  |  |  |  |  |  |  |  |  |
| Sharon et al |  |  |  |  |  |  |  |  |  |  |  |  |  |  |
| Gnarini et al |  |  |  |  |  |  |  |  |  |  |  |  |  |  |
| Armanini et al | 15/17 | 2/17 | 12/17 | 5/17 |  |  |  |  |  |  |  |  |  |  |
| Liebeskind et al |  |  | 11/23 | 12/23 | 10/23 | 0/23 | 13/23 |  |  |  |  |  |  |  |
| Jiang et al |  |  | 1099/3100 | 2001/3100 |  |  |  |  |  |  |  |  |  |  |
| Akarsu et al |  |  |  |  |  |  |  |  |  |  |  |  |  |  |
| Kocak et al |  |  |  |  |  |  |  |  |  |  |  |  |  |  |
| Wang et al |  |  |  |  |  |  |  |  |  |  |  |  | 61/212 | 1904/6291 |
| Song et al |  |  |  |  |  |  |  |  | 97/187 | 81/187 |  | 147/1084 | 40/187 |  |
| Yang et al |  |  |  |  |  |  |  | 898/1558 | 800/1976 |  |  |  | 1461/2466 | 613/1068 |
| Chen et al |  |  |  |  |  |  |  | 127/792 | 111/487 |  | 36/256 | 202/1023 | 57/377 | 181/902 |
| Xu et al |  |  |  |  |  |  |  | 20924/64334 | 13418/36808 | 3313/9289 |  |  |  |  |
| Tian et al |  |  |  |  |  |  |  |  |  |  |  |  |  |  |
| Song et al |  |  |  |  |  |  |  |  |  |  |  |  |  |  |
| Sun et al |  |  |  |  |  |  |  |  |  |  |  |  | 445/3144 | 1587/9554 |
| Fan et al |  |  |  |  |  |  |  |  |  |  |  |  | 204/1000 | 494/2357 |
| Chen et al |  |  |  |  |  |  |  |  |  |  | 496/1824 | 2096/6537 | 1677/2259 | 2218/7087 |
| Guo et al |  |  |  |  |  |  |  |  |  |  |  |  |  |  |
| Kamran et al |  |  |  |  |  |  |  |  |  |  |  |  |  |  |
| Yao et al |  |  |  |  |  |  |  |  |  |  |  |  |  |  |
| Batolotta et al |  |  |  |  |  |  |  |  |  |  |  |  |  |  |
| Acar et al |  |  |  |  |  |  |  |  |  |  |  |  |  |  |
| Zhu et al |  |  | 1768/3514 | 1746/3514 |  |  |  |  |  |  |  |  |  |  |
| Zeng et al |  |  |  |  |  |  |  |  |  |  |  |  |  |  |
| Zhu et al |  |  |  |  |  |  |  | 1548678/4296187 | 1269398/3033502 | 512812/1199245 |  |  |  |  |
| Li et al |  |  |  |  |  |  |  |  |  |  |  |  |  |  |
| Xu et al |  |  |  |  |  |  |  |  |  |  | 366/1237 | 1822/5430 | 383/1404 | 1825/5333 |
| Liu et al |  |  |  |  |  |  |  |  |  |  |  |  |  |  |
| Wang et al |  |  |  |  |  |  |  | 18900/46736 | 1588/4126 | 296/775 | 6910/16686 | 13874/34951 | 5245/12761 | 15539/38876 |
| Lai et al |  |  |  |  |  |  |  |  |  |  |  |  |  |  |
| Kim et al |  |  |  |  |  |  |  |  |  |  |  |  |  |  |
| Guo et al |  |  |  |  |  |  |  |  |  |  |  |  |  |  |
| Zou et al |  |  |  |  |  |  |  | 1111/4462 | 1310/4012 | 540/1770 |  |  |  |  |
| Feng et al |  |  |  |  |  |  |  |  |  |  |  |  |  |  |
| Karger et al |  |  |  |  |  |  |  | 1111/2523 | 1310/2702 | 540/1230 |  |  |  |  |
| Shokri et al |  |  |  |  |  |  |  |  |  |  |  |  |  |  |
| Gao et al |  |  |  |  |  |  |  |  |  |  |  |  |  |  |
| Piciu et al |  |  |  |  |  |  |  |  |  |  |  |  |  |  |
| Piciu et al |  |  |  |  |  |  |  |  |  |  |  |  |  |  |
| Meng et al |  |  |  |  |  |  |  |  |  |  |  |  |  |  |
| Solares et al |  |  |  |  |  |  |  |  |  |  |  |  |  |  |
| Zhang et al |  |  |  |  |  |  |  | 806609/2467806 | 720084/1763291 | 399850/689439 |  |  |  |  |
| Rizza et al |  |  |  |  |  |  |  |  |  |  |  |  |  |  |
| Marwaha et al |  |  |  |  |  |  |  |  |  |  |  |  |  |  |
| Karatoprak et al |  |  |  |  |  |  |  |  |  |  |  |  |  |  |
| Li et al |  |  |  |  |  |  |  |  |  |  |  |  |  |  |
| Li et al |  |  |  |  |  |  |  |  |  |  |  |  |  |  |
| Li et al |  |  |  |  |  |  |  |  |  |  |  |  |  |  |
| Shan et al |  |  |  |  |  |  |  |  |  |  |  |  |  |  |
| Guth et al |  |  |  |  |  |  |  |  |  |  |  |  |  |  |
| Dauksiene et al |  |  |  |  |  |  |  |  |  |  |  |  |  |  |
| Zheng et al |  |  |  |  |  |  |  |  |  |  |  |  |  |  |
| Knudsen et al |  |  |  |  |  |  |  |  |  |  |  |  |  |  |
| Li et al |  |  |  |  |  |  |  |  |  |  |  |  |  |  |
| Suk et al |  |  |  |  |  |  |  |  |  |  |  |  |  |  |
| Panagiotou et al |  |  |  |  |  |  |  |  |  |  |  |  |  |  |
| Liu et al |  |  |  |  |  |  |  |  |  |  |  |  |  |  |
| Wang et al |  |  |  |  |  |  |  |  |  |  |  |  |  |  |
| Shen et al |  |  |  |  |  |  |  |  |  |  |  |  |  |  |
| Lopez et al |  |  |  |  |  |  |  |  |  |  |  |  |  |  |
| Reiner et al |  |  |  |  |  |  |  |  |  |  |  |  |  |  |
| Yin et al |  |  |  |  |  |  |  |  |  |  |  |  |  |  |
| Imani et al |  |  |  |  |  |  |  |  |  |  |  |  |  |  |
| Liu et al | 18466/24010 | 5544/24010 | 12039/24010 | 11971/24010 |  |  |  |  |  |  |  |  |  |  |
| Ding et al |  |  |  |  |  |  |  |  |  |  |  |  |  |  |
| Oh et al |  |  |  |  |  |  |  |  |  |  |  |  |  |  |
| Kim et al |  |  | 552/1082 | 530/1082 |  |  |  |  |  |  |  |  |  |  |
| Zou et al |  |  |  |  |  |  |  |  |  |  |  |  |  |  |
| Kim et al |  |  |  |  |  |  |  |  |  |  |  |  |  |  |
| Delitala et al |  |  | 341/588 | 247/588 |  |  |  |  |  |  |  |  |  |  |
| Du et al |  |  |  |  |  |  |  |  |  |  |  |  |  |  |
| Wang et al |  |  |  |  |  |  |  |  |  |  |  |  |  |  |
| Yan et al |  |  |  |  |  |  |  |  |  |  |  |  |  |  |
| Gao et al |  |  |  |  |  |  |  |  |  |  |  |  |  |  |
| Sahin et al |  |  |  |  |  |  |  |  |  |  |  |  |  |  |
| Kim et al |  |  |  |  |  |  |  |  |  |  |  |  |  |  |
| Kwon et al |  |  |  |  |  |  |  |  |  |  |  |  |  |  |
| Li et al |  |  |  |  |  |  |  |  |  |  |  |  |  |  |
| Wang et al |  |  |  |  |  |  |  |  |  |  |  |  |  |  |
| Pan et al |  |  |  |  |  |  |  |  |  |  |  |  |  |  |
| Krejbjerg et al |  |  |  |  |  |  |  |  |  |  |  |  |  |  |
| Brauer et al |  |  |  |  |  |  |  |  |  |  |  |  |  |  |
| Neuhold et al |  |  |  |  |  |  |  |  |  |  |  |  |  |  |
| de matos et al |  |  |  |  |  |  |  |  |  |  |  |  |  |  |
| Kovacs et al |  |  |  |  |  |  |  |  |  |  |  |  |  |  |
| Szolnok et al |  |  |  |  |  |  |  |  |  |  |  |  |  |  |
| Mitselou et al |  |  |  |  |  |  |  |  |  |  |  |  |  |  |
| Solares et al |  |  |  |  |  |  |  |  |  |  |  |  |  |  |
| Tanriover et al |  |  |  |  |  |  |  |  |  |  |  |  |  |  |
| Mohorea et al |  |  |  |  |  |  |  |  |  |  |  |  |  |  |
| Ramirez et al |  |  |  |  |  |  |  |  |  |  |  |  |  |  |
| Hurtado et al |  |  |  |  |  |  |  |  |  |  |  |  |  |  |
| Kikuchi et al |  |  |  |  |  |  |  |  |  |  |  |  |  |  |

**Supplementary Table 2. Leave one out analysis for detection of heterogeneity.**

resid se z

29 0.5398 0.1841 2.9328

59 0.4298 0.1873 2.2950

86 -0.4220 0.1863 -2.2654

53 -0.4204 0.1865 -2.2545

97 -0.3952 0.1916 -2.0626

100 -0.3421 0.1903 -1.7975

56 -0.3368 0.1883 -1.7889

87 0.3360 0.1889 1.7785

98 -0.3428 0.1943 -1.7643

19 0.3320 0.1884 1.7625

95 -0.3279 0.1914 -1.7136

94 -0.3180 0.1915 -1.6603

65 0.3006 0.1910 1.5738

48 0.2981 0.1961 1.5204

61 0.2688 0.1894 1.4187

39 -0.2652 0.1893 -1.4012

33 0.2622 0.1900 1.3800

96 -0.2641 0.1935 -1.3652

93 -0.2581 0.1934 -1.3348

31 0.2550 0.1916 1.3313

92 -0.2447 0.1952 -1.2539

88 0.2366 0.1898 1.2465

3 0.2366 0.1898 1.2465

14 0.2340 0.1898 1.2327

99 -0.2282 0.1910 -1.1947

66 0.2259 0.1898 1.1901

89 0.2117 0.1903 1.1124

27 0.2092 0.1901 1.1005

47 0.2109 0.1954 1.0796

4 -0.2020 0.1921 -1.0511

44 0.1963 0.1917 1.0238

50 -0.1985 0.1945 -1.0205

11 0.1963 0.1934 1.0147

68 0.1835 0.1909 0.9609

18 -0.1822 0.1907 -0.9553

57 -0.1798 0.1903 -0.9446

58 -0.1794 0.1903 -0.9425

83 -0.1746 0.1907 -0.9159

46 -0.1714 0.1907 -0.8992

10 0.1691 0.1904 0.8880

78 -0.1665 0.1905 -0.8737

85 -0.1607 0.1911 -0.8408

49 -0.1585 0.1908 -0.8310

102 -0.1563 0.1906 -0.8202

64 0.1511 0.1907 0.7924

38 0.1448 0.1906 0.7595

7 -0.1466 0.1954 -0.7504

80 -0.1429 0.1909 -0.7485

13 -0.1377 0.1935 -0.7117

24 -0.1337 0.1907 -0.7007

75 0.1326 0.1908 0.6953

34 0.1319 0.1907 0.6915

45 -0.1261 0.1919 -0.6575

2 0.1256 0.1911 0.6574

51 0.1215 0.1908 0.6368

63 0.1195 0.1910 0.6257

62 -0.1178 0.1909 -0.6168

82 -0.1176 0.1913 -0.6146

77 0.1150 0.1911 0.6020

79 -0.1145 0.1910 -0.5993

43 -0.1106 0.1909 -0.5793

35 0.1101 0.1909 0.5768

22 0.1098 0.1910 0.5747

40 0.1070 0.1911 0.5600

32 0.1065 0.1909 0.5575

37 -0.0988 0.1915 -0.5160

20 -0.0986 0.1914 -0.5149

73 0.0945 0.1910 0.4949

71 0.0897 0.1910 0.4694

101 -0.0858 0.1912 -0.4485

69 -0.0858 0.1912 -0.4485

1 0.0819 0.1910 0.4287

21 0.0809 0.1910 0.4234

55 -0.0736 0.1910 -0.3850

9 0.0741 0.1933 0.3834

70 0.0698 0.1911 0.3652

30 0.0700 0.1920 0.3646

28 -0.0695 0.1934 -0.3591

36 0.0666 0.1912 0.3482

74 0.0620 0.1912 0.3244

42 0.0620 0.1911 0.3243

52 0.0597 0.1932 0.3090

12 -0.0611 0.1990 -0.3071

76 -0.0514 0.1912 -0.2690

6 -0.0510 0.1930 -0.2642

60 0.0502 0.1931 0.2599

72 -0.0497 0.1936 -0.2570

17 0.0385 0.1912 0.2013

84 0.0400 0.1987 0.2010

67 0.0364 0.1912 0.1904

26 0.0359 0.1912 0.1878

90 0.0228 0.1913 0.1192

81 0.0219 0.1912 0.1143

91 0.0219 0.1920 0.1141

5 0.0172 0.1926 0.0892

23 0.0112 0.1913 0.0584

15 0.0106 0.1931 0.0548

8 -0.0078 0.1937 -0.0405

41 -0.0073 0.1914 -0.0382

16 -0.0033 0.1914 -0.0173

54 -0.0017 0.1981 -0.0087

25 -0.0007 0.1914 -0.0037

estimate zval pval ci.lb ci.ub Q Qp tau2 I2 H2

1 0.247381 -11.329789 0.000000 0.213307 0.284927 9359521.297186 0.000000 0.955986 99.997645 42459.095390

2 0.247053 -11.360038 0.000000 0.213046 0.284525 9361188.537718 0.000000 0.953903 99.997650 42556.769675

3 0.246247 -11.452333 0.000000 0.212455 0.283479 9359927.252372 0.000000 0.945755 99.997629 42180.737656

4 0.250013 -11.234401 0.000000 0.215820 0.287637 9361274.150313 0.000000 0.947994 99.997636 42298.375438

5 0.247903 -11.291944 0.000000 0.213751 0.285531 9361311.268042 0.000000 0.957697 99.997660 42730.630478

6 0.248485 -11.260853 0.000000 0.214277 0.286164 9361307.488248 0.000000 0.957651 99.997660 42728.949364

7 0.249384 -11.239527 0.000000 0.215168 0.287051 9361301.538814 0.000000 0.953266 99.997649 42533.785463

8 0.248116 -11.280058 0.000000 0.213942 0.285764 9361311.328468 0.000000 0.957844 99.997660 42737.695828

9 0.247459 -11.325172 0.000000 0.213376 0.285013 9361305.853240 0.000000 0.956204 99.997656 42664.309348

10 0.246730 -11.393536 0.000000 0.212800 0.284118 9359014.298589 0.000000 0.951193 99.997641 42383.104763

11 0.246570 -11.414308 0.000000 0.212690 0.283902 9361280.080778 0.000000 0.949360 99.997639 42359.136308

12 0.248586 -11.262907 0.000000 0.214390 0.286249 9361310.091281 0.000000 0.956882 99.997658 42695.167322

13 0.249306 -11.237988 0.000000 0.215080 0.286986 9361296.713976 0.000000 0.954038 99.997651 42568.077902

14 0.246265 -11.449920 0.000000 0.212468 0.283504 9359900.269790 0.000000 0.945990 99.997630 42190.502014

15 0.247960 -11.288744 0.000000 0.213802 0.285593 9361311.479759 0.000000 0.957753 99.997660 42733.378043

16 0.248067 -11.280714 0.000000 0.213892 0.285717 9361310.842836 0.000000 0.958009 99.997660 42739.718125

17 0.247722 -11.303105 0.000000 0.213592 0.285328 9361281.845035 0.000000 0.957372 99.997658 42700.633860

18 0.249807 -11.230767 0.000000 0.215591 0.287463 9361163.698192 0.000000 0.950302 99.997641 42399.809792

19 0.245574 -11.552068 0.000000 0.212022 0.282532 9359783.977392 0.000000 0.935364 99.997603 41725.126959

20 0.248924 -11.243340 0.000000 0.214692 0.286623 9361264.697081 0.000000 0.956422 99.997657 42672.478452

21 0.247389 -11.329129 0.000000 0.213313 0.284936 9358373.500008 0.000000 0.956026 99.997637 42327.667285

22 0.247170 -11.348724 0.000000 0.213138 0.284670 9361141.600955 0.000000 0.954731 99.997652 42589.095996

23 0.247945 -11.288046 0.000000 0.213784 0.285582 9361310.721382 0.000000 0.957876 99.997660 42727.204708

24 0.249278 -11.234600 0.000000 0.215041 0.286973 9360535.986465 0.000000 0.954556 99.997651 42570.974708

25 0.248045 -11.282012 0.000000 0.213872 0.285693 9361311.179909 0.000000 0.957991 99.997660 42738.534620

26 0.247743 -11.301608 0.000000 0.213609 0.285352 9361274.976037 0.000000 0.957435 99.997658 42696.105703

27 0.246442 -11.427395 0.000000 0.212591 0.283741 9359611.426435 0.000000 0.948146 99.997635 42277.139036

28 0.248652 -11.254587 0.000000 0.214436 0.286337 9361306.022569 0.000000 0.957216 99.997659 42709.726718

29 0.244060 -11.866263 0.000000 0.211271 0.280131 9360556.991470 0.000000 0.898896 99.997507 40106.394807

30 0.247480 -11.322327 0.000000 0.213391 0.285042 9361299.269361 0.000000 0.956400 99.997657 42671.945298

31 0.246142 -11.467965 0.000000 0.212388 0.283331 9361228.246864 0.000000 0.944224 99.997626 42129.609816

32 0.247194 -11.346390 0.000000 0.213157 0.284700 9360893.648528 0.000000 0.954896 99.997651 42579.868565

33 0.246073 -11.476484 0.000000 0.212339 0.283239 9361019.376364 0.000000 0.943351 99.997624 42088.479488

34 0.247003 -11.364675 0.000000 0.213007 0.284464 8342834.330482 0.000000 0.953553 99.995974 24837.680001

35 0.247166 -11.348930 0.000000 0.213134 0.284666 8915190.419800 0.000000 0.954719 99.996323 27195.353551

36 0.247500 -11.319991 0.000000 0.213405 0.285069 9361204.945458 0.000000 0.956553 99.997656 42662.559975

37 0.248926 -11.243315 0.000000 0.214694 0.286625 9361267.214489 0.000000 0.956409 99.997657 42672.040096

38 0.246908 -11.374401 0.000000 0.212934 0.284344 9356994.191849 0.000000 0.952787 99.997639 42363.400888

39 0.250901 -11.249498 0.000000 0.216805 0.288385 9311676.405787 0.000000 0.936832 99.997593 41540.721149

40 0.247191 -11.346762 0.000000 0.213155 0.284696 9361151.012319 0.000000 0.954869 99.997652 42595.285121

41 0.248100 -11.278736 0.000000 0.213922 0.285755 9361309.988142 0.000000 0.958033 99.997660 42740.608139

42 0.247536 -11.317139 0.000000 0.213435 0.285111 9361188.845162 0.000000 0.956707 99.997656 42663.354751

43 0.249043 -11.239626 0.000000 0.214807 0.286743 9361022.960202 0.000000 0.955903 99.997655 42640.234407

44 0.246551 -11.415281 0.000000 0.212672 0.283881 9361245.142232 0.000000 0.949269 99.997639 42354.385341

45 0.249196 -11.237513 0.000000 0.214963 0.286889 9361278.577089 0.000000 0.954941 99.997653 42607.735887

46 0.249686 -11.230695 0.000000 0.215462 0.287354 9361097.544053 0.000000 0.951426 99.997644 42448.653522

47 0.246491 -11.425409 0.000000 0.212637 0.283792 9361289.429878 0.000000 0.948351 99.997637 42314.321956

48 0.245913 -11.504493 0.000000 0.212248 0.283000 9361278.657178 0.000000 0.940548 99.997617 41966.271649

49 0.249542 -11.231529 0.000000 0.215312 0.287222 9361111.113412 0.000000 0.952628 99.997647 42501.894488

50 0.249936 -11.237775 0.000000 0.215747 0.287555 9361294.156179 0.000000 0.948465 99.997637 42319.590888

51 0.247081 -11.357065 0.000000 0.213067 0.284560 9010722.694063 0.000000 0.954129 99.996725 30536.799753

52 0.247569 -11.316309 0.000000 0.213468 0.285144 9361307.906867 0.000000 0.956685 99.997657 42685.760295

53 0.254216 -11.542782 0.000000 0.221146 0.290387 9360627.816735 0.000000 0.860323 99.997395 38386.288221

54 0.248087 -11.285766 0.000000 0.213928 0.285718 9361311.480749 0.000000 0.957471 99.997659 42721.414823

55 0.248686 -11.250626 0.000000 0.214461 0.286382 9359596.112809 0.000000 0.957350 99.997650 42561.273152

56 0.252112 -11.316007 0.000000 0.218280 0.289247 9360607.562667 0.000000 0.914682 99.997550 40810.632274

57 0.249784 -11.230114 0.000000 0.215565 0.287445 9359847.603455 0.000000 0.950572 99.997641 42393.715039

58 0.249779 -11.230117 0.000000 0.215560 0.287441 9359852.479680 0.000000 0.950616 99.997641 42395.606254

59 0.244893 -11.677232 0.000000 0.211645 0.281499 9360893.721924 0.000000 0.921253 99.997567 41104.012800

60 0.247643 -11.310616 0.000000 0.213529 0.285230 9361308.932067 0.000000 0.956966 99.997658 42698.277243

61 0.246021 -11.483550 0.000000 0.212303 0.283168 9360409.761340 0.000000 0.942626 99.997622 42050.118198

62 0.249115 -11.237978 0.000000 0.214878 0.286813 9361092.868000 0.000000 0.955519 99.997654 42626.934911

63 0.247097 -11.355634 0.000000 0.213081 0.284581 9361166.472189 0.000000 0.954232 99.997651 42569.705775

64 0.246862 -11.379220 0.000000 0.212899 0.284287 9360632.819640 0.000000 0.952396 99.997646 42473.549490

65 0.245825 -11.513886 0.000000 0.212181 0.282888 9361202.338229 0.000000 0.939497 99.997614 41918.779600

66 0.246322 -11.442504 0.000000 0.212506 0.283580 9333346.708453 0.000000 0.946709 99.997610 41843.655845

67 0.247739 -11.301867 0.000000 0.213605 0.285347 9361137.131301 0.000000 0.957426 99.997653 42609.446948

68 0.246633 -11.405024 0.000000 0.212730 0.283990 9361176.713812 0.000000 0.950189 99.997641 42393.789090

69 0.248801 -11.246870 0.000000 0.214572 0.286499 9361242.688202 0.000000 0.956942 99.997658 42693.422461

70 0.247475 -11.321996 0.000000 0.213384 0.285038 9359629.322707 0.000000 0.956444 99.997643 42418.814067

71 0.247322 -11.334936 0.000000 0.213259 0.284855 9360905.018263 0.000000 0.955663 99.997653 42603.840056

72 0.248475 -11.261855 0.000000 0.214270 0.286152 9361308.501481 0.000000 0.957618 99.997660 42727.614791

73 0.247284 -11.338178 0.000000 0.213229 0.284810 9359024.505174 0.000000 0.955453 99.997644 42444.798355

74 0.247536 -11.317165 0.000000 0.213435 0.285111 9361220.261712 0.000000 0.956705 99.997656 42669.466187

75 0.246998 -11.365233 0.000000 0.213003 0.284458 9360241.597593 0.000000 0.953510 99.997647 42500.714463

76 0.248484 -11.259011 0.000000 0.214271 0.286170 9361255.229846 0.000000 0.957817 99.997660 42726.911886

77 0.247131 -11.352425 0.000000 0.213108 0.284622 9361193.167919 0.000000 0.954465 99.997652 42580.996559

78 0.249632 -11.230638 0.000000 0.215405 0.287306 9360637.684330 0.000000 0.951918 99.997645 42462.807552

79 0.249081 -11.238805 0.000000 0.214845 0.286781 9361152.256054 0.000000 0.955693 99.997655 42636.421057

80 0.249374 -11.233462 0.000000 0.215139 0.287063 9361164.926807 0.000000 0.953882 99.997650 42558.140464

81 0.247857 -11.293750 0.000000 0.213707 0.285482 9361299.976542 0.000000 0.957718 99.997659 42707.759684

82 0.249111 -11.238488 0.000000 0.214876 0.286809 9361245.522446 0.000000 0.955497 99.997654 42631.274463

83 0.249721 -11.230626 0.000000 0.215500 0.287387 9361109.239911 0.000000 0.951107 99.997643 42434.689610

84 0.247761 -11.306898 0.000000 0.213643 0.285350 9361311.158017 0.000000 0.956905 99.997658 42696.145407

85 0.249563 -11.231878 0.000000 0.215335 0.287239 9361222.352532 0.000000 0.952419 99.997647 42494.623428

86 0.254364 -11.562307 0.000000 0.221357 0.290457 140516.786344 0.000000 0.855954 99.996694 30250.137755

87 0.245556 -11.555605 0.000000 0.212012 0.282504 9360934.808528 0.000000 0.934988 99.997603 41716.055297

88 0.246247 -11.452333 0.000000 0.212455 0.283479 9359927.252372 0.000000 0.945755 99.997629 42180.737656

89 0.246427 -11.429459 0.000000 0.212581 0.283720 9360938.917184 0.000000 0.947953 99.997635 42290.866228

90 0.247850 -11.294310 0.000000 0.213701 0.285474 9361306.201318 0.000000 0.957695 99.997659 42721.292247

91 0.247861 -11.294143 0.000000 0.213713 0.285485 9361310.614271 0.000000 0.957667 99.997660 42728.371652

92 0.250434 -11.248279 0.000000 0.216306 0.287968 9361293.689464 0.000000 0.942388 99.997622 42048.520716

93 0.250647 -11.250792 0.000000 0.216539 0.288151 9361285.217109 0.000000 0.939761 99.997615 41931.279909

94 0.251506 -11.287100 0.000000 0.217554 0.288800 9361270.942410 0.000000 0.925825 99.997579 41309.521181

95 0.251649 -11.296140 0.000000 0.217732 0.288898 9361270.791897 0.000000 0.923082 99.997572 41187.128190

96 0.250716 -11.253417 0.000000 0.216620 0.288204 9361285.588571 0.000000 0.938731 99.997613 41885.355419

97 0.252230 -11.354796 0.000000 0.218516 0.289222 9361286.654996 0.000000 0.909734 99.997536 40591.662786

98 0.251517 -11.301896 0.000000 0.217609 0.288759 9361291.594046 0.000000 0.924445 99.997576 41248.003890

99 0.250351 -11.237470 0.000000 0.216187 0.287931 9361239.847729 0.000000 0.944217 99.997626 42129.698128

100 0.251952 -11.313657 0.000000 0.218104 0.289111 9361256.060311 0.000000 0.917268 99.997557 40927.703556

101 0.248801 -11.246870 0.000000 0.214572 0.286499 9361242.688202 0.000000 0.956942 99.997658 42693.422461

102 0.249520 -11.231486 0.000000 0.215288 0.287202 9360805.982415 0.000000 0.952826 99.997647 42504.869429

**Supplementary Table 3. Univariate analysis for the covariates.**

| Country | Mixed-Effects Model (k = 98; tau^2 estimator: DL)  tau^2 (estimated amount of residual heterogeneity): 0.0279 (SE = 0.0155)  tau (square root of estimated tau^2 value): 0.1669  I^2 (residual heterogeneity / unaccounted variability): 99.32%  H^2 (unaccounted variability / sampling variability): 146.94  R^2 (amount of heterogeneity accounted for): 0.00%  Test for Residual Heterogeneity:  QE(df = 72) = 10579.6810, p-val < .0001  Test of Moderators (coefficients 2:26):  F(df1 = 25, df2 = 72) = 1.1790, p-val = 0.2883  Model Results:  estimate se tval df pval ci.lb  intrcpt 0.0850 0.1705 0.4986 72 0.6196 -0.2549  countryBrazil -0.0070 0.2357 -0.0297 72 0.9764 -0.4769  countryCameroon 0.1980 0.2360 0.8391 72 0.4042 -0.2724  countryCyprus 0.3820 0.2717 1.4062 72 0.1640 -0.1595  countryDenmark 0.1447 0.2001 0.7230 72 0.4720 -0.2543  countryEthiopia 0.2490 0.2437 1.0218 72 0.3103 -0.2368  countryGermany 0.3460 0.1877 1.8435 72 0.0694 -0.0281  countryGhana 0.0270 0.2326 0.1161 72 0.9079 -0.4367  countryGreece 0.2124 0.2081 1.0205 72 0.3109 -0.2025  countryGuatemala -0.0650 0.2281 -0.2850 72 0.7764 -0.5196  countryHungary -0.0375 0.2024 -0.1853 72 0.8535 -0.4411  countryIndia -0.0690 0.2250 -0.3067 72 0.7600 -0.5175  countryIran 0.1068 0.2056 0.5194 72 0.6051 -0.3030  countryItaly 0.2066 0.1871 1.1046 72 0.2730 -0.1663  countryJapan 0.0590 0.2254 0.2618 72 0.7942 -0.3903  countryKorea 0.2145 0.1784 1.2022 72 0.2332 -0.1412  countryLithuania 0.2270 0.2414 0.9402 72 0.3502 -0.2543  countryMainland China 0.2348 0.1719 1.3658 72 0.1763 -0.1079  countryMexcio 0.1110 0.2266 0.4899 72 0.6257 -0.3407  countryMexico 0.0342 0.2009 0.1701 72 0.8654 -0.3664  countryNigeria 0.1390 0.2376 0.5851 72 0.5603 -0.3346  countryPakistan 0.1483 0.2091 0.7091 72 0.4806 -0.2685  countryPoland 0.0630 0.2475 0.2546 72 0.7998 -0.4304  countryRomania 0.1535 0.2126 0.7223 72 0.4725 -0.2702  countryTurkey 0.1808 0.1850 0.9776 72 0.3316 -0.1879  countryUSA 0.0498 0.2083 0.2392 72 0.8116 -0.3654  ci.ub  intrcpt 0.4249  countryBrazil 0.4629  countryCameroon 0.6684  countryCyprus 0.9235  countryDenmark 0.5437  countryEthiopia 0.7348  countryGermany 0.7202 .  countryGhana 0.4907  countryGreece 0.6273  countryGuatemala 0.3896  countryHungary 0.3661  countryIndia 0.3795  countryIran 0.5166  countryItaly 0.5795  countryJapan 0.5083  countryKorea 0.5701  countryLithuania 0.7083  countryMainland China 0.5775  countryMexcio 0.5627  countryMexico 0.4347  countryNigeria 0.6126  countryPakistan 0.5651  countryPoland 0.5564  countryRomania 0.5773  countryTurkey 0.5496  countryUSA 0.4650 |
| --- | --- |
| Development | Mixed-Effects Model (k = 98; tau^2 estimator: DL)  tau^2 (estimated amount of residual heterogeneity): 0.0263 (SE = 0.0136)  tau (square root of estimated tau^2 value): 0.1623  I^2 (residual heterogeneity / unaccounted variability): 99.19%  H^2 (unaccounted variability / sampling variability): 124.00  R^2 (amount of heterogeneity accounted for): 4.00%  Test for Residual Heterogeneity:  QE(df = 96) = 11903.8896, p-val < .0001  Test of Moderators (coefficient 2):  F(df1 = 1, df2 = 96) = 0.6116, p-val = 0.4361  Model Results:  estimate se tval df pval ci.lb ci.ub  intrcpt 0.2606 0.0295 8.8235 96 <.0001 0.2019 0.3192  developDeveloping 0.0275 0.0352 0.7820 96 0.4361 -0.0423 0.0974 |
| Diagnostic technique | Mixed-Effects Model (k = 98; tau^2 estimator: DL)  tau^2 (estimated amount of residual heterogeneity): 0.0260 (SE = 0.0134)  tau (square root of estimated tau^2 value): 0.1611  I^2 (residual heterogeneity / unaccounted variability): 99.22%  H^2 (unaccounted variability / sampling variability): 127.63  R^2 (amount of heterogeneity accounted for): 0.28%  Test for Residual Heterogeneity:  QE(df = 96) = 12252.7378, p-val < .0001  Test of Moderators (coefficient 2):  F(df1 = 1, df2 = 96) = 24.7693, p-val < .0001  Model Results:  estimate se tval df pval ci.lb ci.ub  intrcpt 0.0623 0.0460 1.3528 96 0.1793 -0.0291 0.1536  diagnosticUS 0.2412 0.0485 4.9769 96 <.0001 0.1450 0.3373 *** |
| Country income assessed by World Bank | Mixed-Effects Model (k = 98; tau^2 estimator: DL)  tau^2 (estimated amount of residual heterogeneity): 0.0275 (SE = 0.0138)  tau (square root of estimated tau^2 value): 0.1659  I^2 (residual heterogeneity / unaccounted variability): 99.21%  H^2 (unaccounted variability / sampling variability): 125.98  R^2 (amount of heterogeneity accounted for): 8.00%  Test for Residual Heterogeneity:  QE(df = 95) = 11968.2351, p-val < .0001  Test of Moderators (coefficients 2:3):  F(df1 = 2, df2 = 95) = 1.0237, p-val = 0.3632  Model Results:  estimate se tval df pval ci.lb ci.ub  intrcpt 0.2626 0.0289 9.1019 95 <.0001 0.2054 0.3199  incomeLower-Middle -0.0634 0.0797 -0.7959 95 0.4281 -0.2216 0.0948  incomeUpper-Middle 0.0315 0.0351 0.8982 95 0.3713 -0.0382 0.1012    intrcpt ***  incomeLower-Middle  incomeUpper-Middle |
| Quality score | Mixed-Effects Model (k = 98; tau^2 estimator: DL)  tau^2 (estimated amount of residual heterogeneity): 0.0261 (SE = 0.0133)  tau (square root of estimated tau^2 value): 0.1615  I^2 (residual heterogeneity / unaccounted variability): 99.14%  H^2 (unaccounted variability / sampling variability): 116.50  R^2 (amount of heterogeneity accounted for): 18.00%  Test for Residual Heterogeneity:  QE(df = 96) = 11184.0324, p-val < .0001  Test of Moderators (coefficient 2):  F(df1 = 1, df2 = 96) = 0.1170, p-val = 0.7330  Model Results:  estimate se tval df pval ci.lb ci.ub  intrcpt 0.2995 0.0594 5.0400 96 <.0001 0.1815 0.4174 ***  quality -0.0176 0.0515 -0.3421 96 0.7330 -0.1199 0.0847 |
| Study size | Mixed-Effects Model (k = 98; tau^2 estimator: DL)  tau^2 (estimated amount of residual heterogeneity): 0.0275 (SE = 0.0137)  tau (square root of estimated tau^2 value): 0.1659  I^2 (residual heterogeneity / unaccounted variability): 99.22%  H^2 (unaccounted variability / sampling variability): 127.80  R^2 (amount of heterogeneity accounted for): 8.00%  Test for Residual Heterogeneity:  QE(df = 96) = 12268.6673, p-val < .0001  Test of Moderators (coefficient 2):  F(df1 = 1, df2 = 96) = 0.1708, p-val = 0.6803  Model Results:  estimate se tval df pval ci.lb ci.ub  intrcpt 0.2596 0.0519 5.0021 96 <.0001 0.1566 0.3626 ***  size 0.0172 0.0417 0.4132 96 0.6803 -0.0656 0.1000 |

**Supplementary Table 4. Results of multivariable meta-regression.**

Multimodel Inference: Final Results

--------------------------

- Number of fitted models: 64

- Full formula: ~ country + develop + size + quality + income + diagnostic

- Coefficient significance test: knha

- Interactions modeled: no

- Evaluation criterion: AICc

Best 5 Models

--------------------------

Global model call: metafor::rma(yi = TE, sei = seTE, mods = form, data = glm.data,

method = method, test = test)

---

Model selection table

(Intrc) devlp dgnst incom qulty size df logLik AICc delta weight

5 + + 3 51.161 -96.1 0.00 0.672

21 + + 0.02325 4 50.338 -92.3 3.81 0.100

7 + + + 4 50.245 -92.1 4.00 0.091

37 + + -0.01159 4 50.159 -91.9 4.17 0.084

13 + + + 5 50.818 -91.0 5.07 0.053

Models ranked by AICc(x)

Multimodel Inference Coefficients

--------------------------

Estimate Std. Error z value Pr(>|z|)

intrcpt 5.763964e-02 5.363062e-02 1.074752e+00 0.2824856

diagnosticUS 2.402078e-01 4.976227e-02 4.827106e+00 0.0000014

quality 3.114190e-03 1.753490e-02 1.775996e-01 0.8590374

developDeveloping 6.632995e-04 1.556284e-02 4.262071e-02 0.9660039

size -1.459537e-03 1.232428e-02 1.184278e-01 0.9057287

incomeLower-Middle -6.042888e-03 3.068083e-02 1.969597e-01 0.8438591

incomeUpper-Middle 2.901217e-03 1.620535e-02 1.790284e-01 0.8579154

countryBrazil 1.107388e-24 5.333348e-12 2.076346e-13 1.0000000

countryCameroon 9.472730e-24 6.271300e-12 1.510489e-12 1.0000000

countryCyprus 9.472737e-23 8.208072e-12 1.154076e-11 1.0000000

countryDenmark -2.106503e-23 5.540937e-12 3.801709e-12 1.0000000

countryEthiopia 2.992938e-23 6.468213e-12 4.627148e-12 1.0000000

countryGermany 7.775698e-23 6.350695e-12 1.224385e-11 1.0000000

countryGhana -7.383897e-23 7.025378e-12 1.051032e-11 1.0000000

countryGreece 5.976458e-23 5.542457e-12 1.078305e-11 1.0000000

countryGuatemala -3.166819e-23 5.175130e-12 6.119303e-12 1.0000000

countryHungary -1.821198e-23 4.524297e-12 4.025372e-12 1.0000000

countryIndia -1.206104e-22 8.115432e-12 1.486186e-11 1.0000000

countryIran -3.530378e-23 5.940026e-12 5.943371e-12 1.0000000

countryItaly 7.734011e-24 5.280280e-12 1.464697e-12 1.0000000

countryJapan -6.276628e-23 6.530469e-12 9.611298e-12 1.0000000

countryKorea -2.273579e-24 5.098129e-12 4.459635e-13 1.0000000

countryLithuania 1.921091e-23 6.330606e-12 3.034609e-12 1.0000000

countryMainland China 2.944316e-23 5.330071e-12 5.523972e-12 1.0000000

countryMexcio -3.291392e-23 6.218352e-12 5.293030e-12 1.0000000

countryMexico -2.533609e-23 4.999228e-12 5.068001e-12 1.0000000

countryNigeria -1.927224e-23 6.354729e-12 3.032740e-12 1.0000000

countryPakistan -1.485661e-23 5.836176e-12 2.545606e-12 1.0000000

countryPoland -6.081747e-23 6.974987e-12 8.719367e-12 1.0000000

countryRomania 4.644442e-23 5.374194e-12 8.642118e-12 1.0000000

countryTurkey 1.903534e-23 5.068727e-12 3.755447e-12 1.0000000

countryUSA -2.233394e-23 5.003619e-12 4.463557e-12 1.0000000

Predictor Importance

--------------------------

model importance

1 diagnostic 9.997713e-01

2 quality 1.294783e-01

3 develop 1.200784e-01

4 size 1.114237e-01

5 income 7.555204e-02

6 country 4.872029e-22

**Supplementary figure 1. Result of build-in function in metaphor.**


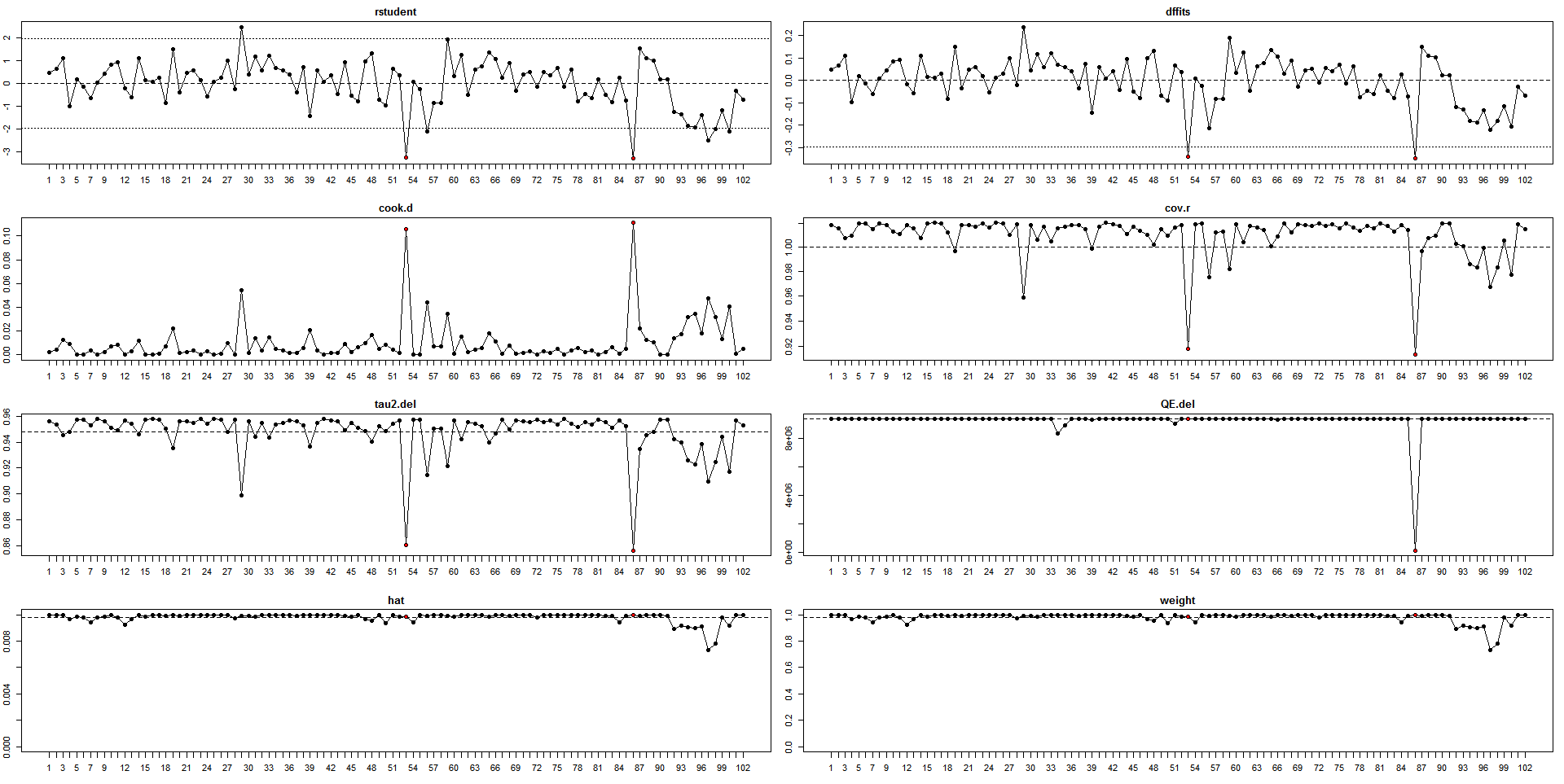


**Supplementary Figure 2. Pooled prevalence of thyroid nodules in individuals smoke or not.**


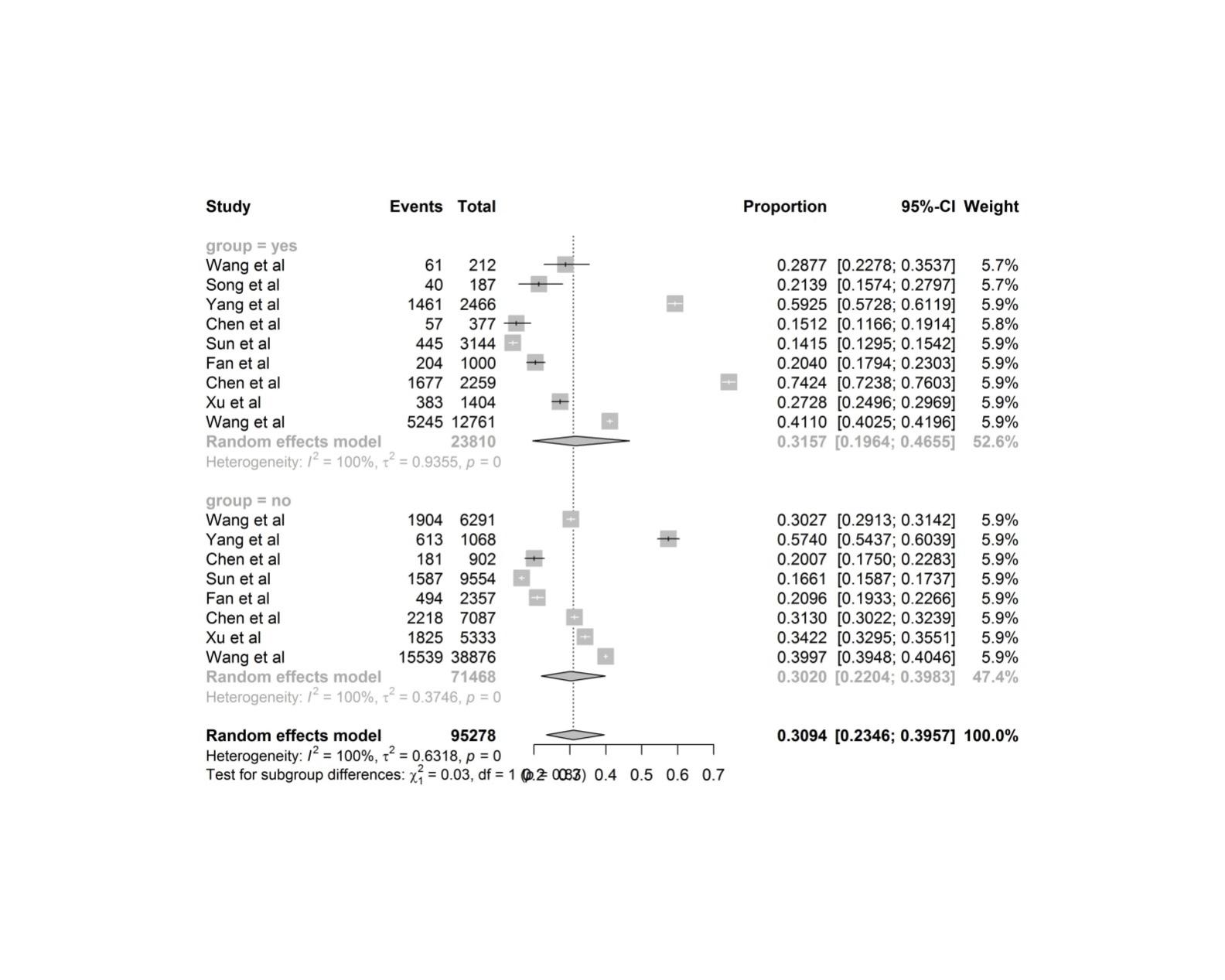


**Supplementary Figure 3. Pooled prevalence of thyroid nodules in individuals consume alcohol or not.**


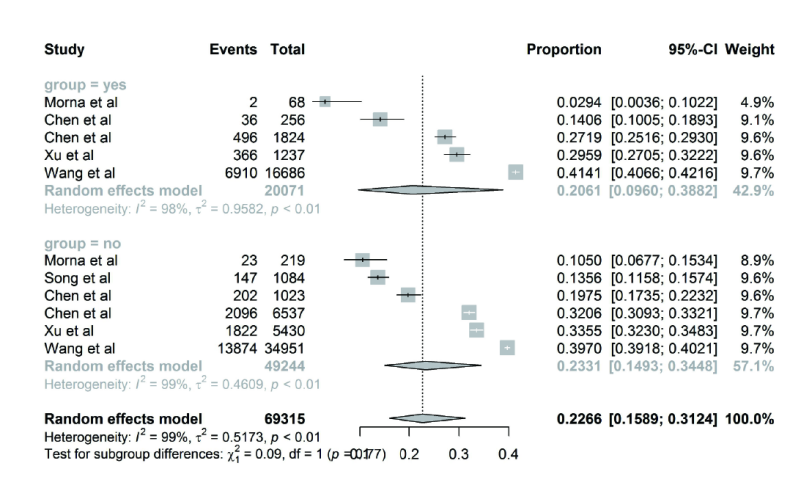


**Supplementary Figure 4. Pooled prevalence of solitary and multiple thyroid nodules.**


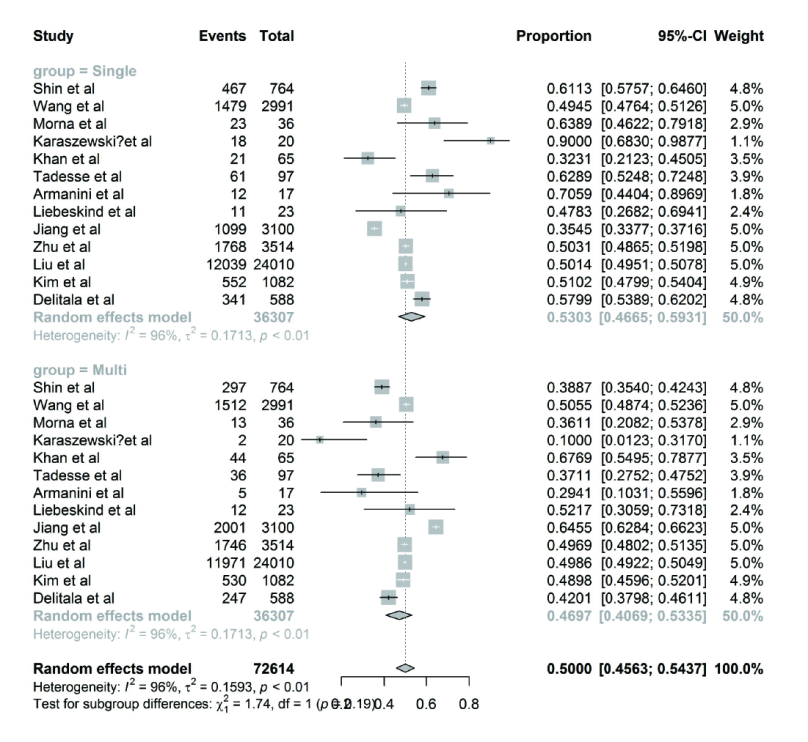


**Supplementary Figure 5. Pooled prevalence of size of thyroid nodules.**


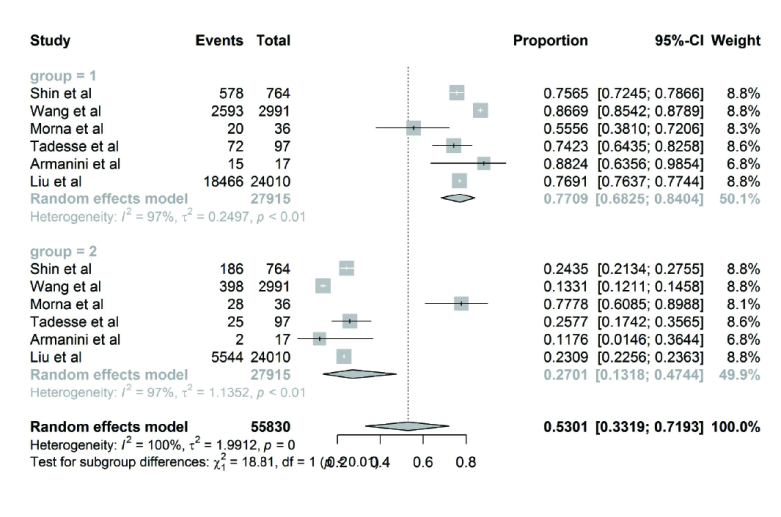


**Supplementary Figure 6. Pooled prevalence of subtypes of thyroid nodules.**


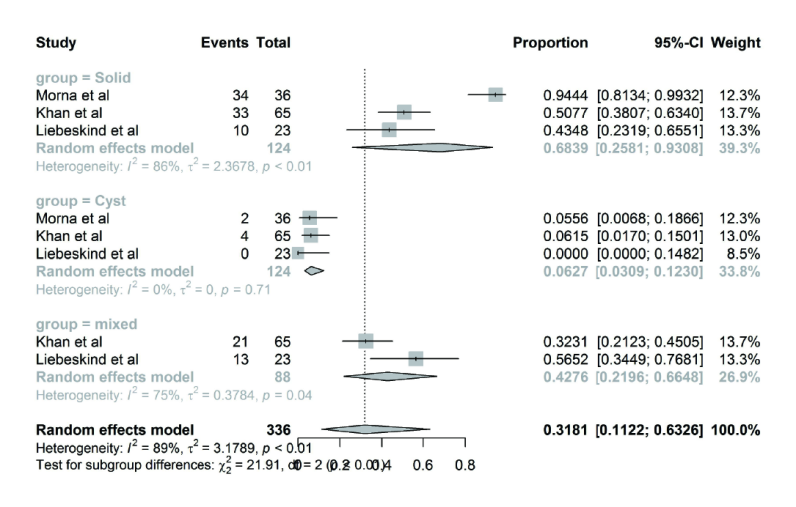

Supplement: Supplementary file 1 [file DataSheet_1.docx]
